# Adaptive Reinforced Elite Gray Wolf Optimization for Robotic Path Planning and Football Pass-Quality Prediction

**DOI:** 10.3390/biomimetics11100748

**Published:** 2026-10-16

**Authors:** Sheng Liu, Huijuan Wang, Yijuan Yuan, Min Zhou, Yonghong Deng

**Affiliations:** 1 College of Sports Science, Guangzhou College of Applied Science and Technology, Zhaoqing 511370, China; liusheng@gzasc.edu.cn (S.L.); zhoumin@gzasc.edu.cn (M.Z.); 2 Department of Physical Education, Liaocheng University Dongchang College, Liaocheng 252000, China; 1120111@lcudcc.edu.cn; 3 School of Science, Key Laboratory of High Performance Scientific Computation, Xihua University, Chengdu 610039, China

**Keywords:** adaptive gray wolf optimizer, swarm intelligence, robotic path planning, hyperparameter optimization, football pass-quality prediction

## Abstract

Complex optimization problems in engineering and data-driven modeling require effective exploration, stable convergence, and adaptability across different decision representations. This study proposes an Adaptive Reinforced Elite Gray Wolf Optimizer (AREGWO) that combines opposition-based initialization, nonlinear convergence control, reinforced elite guidance, adaptive weak perturbation, stagnation-aware partial restart, and greedy elite inheritance. AREGWO is evaluated through CEC2022 benchmark optimization, synthetic 2D constrained robotic path planning, and football pass-quality prediction. On CEC2022, AREGWO ranks second among nine algorithms in the Friedman test and significantly outperforms seven of eight competitors in the Wilcoxon signed-rank test, while L-SHADE remains the strongest baseline. Component-wise ablation shows that the six mechanisms provide complementary and problem-dependent contributions. In robotic path planning, 30 independent runs on six random maps yield a six-map average path length of 134.38 m, which is 3.61–5.12% shorter than those obtained by the four population-based baselines and 1.08% shorter than A*, with a 100% success rate in all tested environments. For football pass-quality prediction, AREGWO is coupled with TabM for outer-level hyperparameter optimization of a predefined pass-quality reference score. AREGWO-TabM achieves an RMSE of 0.445, an MSE of 0.198, and an R^2^ of 0.920, providing the best RMSE, MSE, and R^2^ among the evaluated neural models, although ResNet attains a lower MAE. These results demonstrate the competitive search capability of AREGWO across the numerical, synthetic spatial, and model-level optimization settings evaluated in this study.

## 1. Introduction

Complex optimization problems are widely encountered in engineering design, intelligent robotics, data-driven modeling, and decision-support systems. Such problems often involve nonlinear objective functions, multimodal search landscapes, coupled decision variables, and complicated constraints, making conventional deterministic optimization difficult to apply efficiently. Population-based metaheuristic algorithms provide an alternative by exploring candidate solutions without requiring gradient information or an explicit analytical representation of the objective landscape. Recent studies have therefore focused on improving the balance between global exploration and local exploitation in swarm-based optimization. Yu et al. developed a multi-strategy adaptive Gray Wolf Optimization method for engineering and high-dimensional problems [1], while Qiu et al. introduced an improved GWO for functional optimization and engineering design [2]. Adaptive self-learning mechanisms have also been investigated to strengthen global search and convergence behavior [3], and multi-strategy GWO has been extended to engineering optimization and data-driven prediction problems [4]. These studies demonstrate the continuing interest in improving the robustness and application capability of bio-inspired swarm optimizers.

These developments are rooted in the Gray Wolf Optimizer (GWO), originally proposed by Mirjalili et al. based on the social hierarchy and cooperative hunting behavior of gray wolves [5]. In the canonical GWO, dominant wolves guide population movement, while a convergence-control mechanism regulates the transition from exploration to exploitation. Although its compact structure and limited number of control parameters make GWO attractive for general optimization, the algorithm may still suffer from premature convergence, insufficient population diversity, and excessive dependence on elite individuals in complex search landscapes. To alleviate these limitations, Zhang et al. introduced scale-free network topology to diversify information exchange within the wolf population [6], while Zhao et al. developed an adaptive mechanism based on transfer-function inertia weighting [7]. More recently, multi-population learning combined with Laplace mutation has been used to enhance diversity and reduce stagnation [8], whereas adaptive opposition strategies have been introduced to improve population initialization and search efficiency in complex engineering problems [9]. These advances indicate that population initialization, adaptive convergence, elite information utilization, and stagnation recovery are important factors governing GWO performance.

An important question is whether such improvements remain effective when the representation of candidate solutions and optimization objectives change substantially. Robotic path planning provides an appropriate engineering setting for examining this issue because a feasible route must simultaneously account for travel distance, obstacle avoidance, directional changes, and safety requirements. Zhao et al. applied a golden-sine GWO to obstacle-crossing robot path planning [10], while Chen et al. combined GWO with an artificial potential field for unmanned aerial vehicle path planning [11]. Dynamic weighting and logistic mapping have also been introduced to improve safe UAV low-altitude penetration paths [12], and improved GWO strategies have been investigated for general UAV path-planning tasks [13]. For mobile robots, GWO has been integrated with the Dynamic Window Approach to improve route generation and local motion adjustment [14], whereas dual mutation and chaotic adaptive neighborhood mechanisms have been incorporated into GWO for engineering optimization and path planning [15]. Related work has further combined chaotic GWO with differential evolution for unmanned marine vehicle navigation [16]. Collectively, these studies show that path planning provides a practical test of whether an optimizer can translate numerical search capability into feasible spatial decisions under geometric constraints.

A structurally different challenge arises in data-driven sports modeling. Artificial intelligence is increasingly used in sports analytics to characterize performance patterns, tactical behavior, and decision processes from structured data [17]. In football, research has progressively moved from aggregate match outcomes toward more detailed representations of passing actions and event sequences. Merlin et al. used machine learning to distinguish football passes with different levels of difficulty and to characterize passing performance [18], while Mendes-Neves et al. investigated large event models for soccer event representation and prediction [19]. Machine- and deep-learning methods have also been applied to soccer outcome prediction [20], and tactical contextualization has been combined with machine learning to support more detailed interpretation of match behavior [21]. At the event level, Transformer-based spatio-temporal point-process modeling has been developed for football event analysis [22], while counterattack detection and decision evaluation have been formulated within an artificial-intelligence framework [23]. Passing-network information has also been exploited for predictive match modeling [24], and convolutional methods have been developed for sequential football event forecasting [25]. More recently, event and tracking information have been used to automate assist identification [26]. These developments reflect a broader transition from coarse match-level prediction toward fine-grained modeling of individual football actions and contextual decision processes.

Football event data are predominantly represented by heterogeneous tabular variables, including spatial coordinates, contextual indicators, pressure states, temporal information, and historical interactions. Their nonlinear relationships can be modeled using neural architectures, but predictive performance may depend strongly on learning rate, regularization, embedding dimension, hidden dimension, dropout, and ensemble configuration. Comparative research on tabular deep learning has shown that increasing architectural complexity does not necessarily guarantee superior predictive performance, highlighting the importance of appropriate model configuration [27]. Hyperparameter selection can therefore be viewed as an outer optimization problem in which candidate configurations are evaluated according to validation performance. This characteristic provides a natural connection between swarm intelligence and neural tabular learning: the prediction model performs supervised learning at the inner level, while a population-based optimizer searches the external hyperparameter space.

Despite the progress described above, two issues remain relevant. First, many GWO variants are evaluated primarily on numerical benchmarks and a particular engineering application, making it difficult to determine whether the same search mechanism can remain effective when transferred between fundamentally different optimization representations. Robotic path planning and neural-model hyperparameter optimization provide two complementary settings for such an assessment. In the former, candidate solutions directly describe spatial routes and their quality is determined by geometric feasibility and path characteristics. In the latter, candidate solutions represent model configurations whose quality can only be determined after neural-network training and validation. Second, standard GWO still relies heavily on a relatively simple leader-guided search mechanism, which may limit population diversity and recovery from stagnation when the search landscape becomes complex. A unified optimization framework capable of maintaining effective exploration, exploiting high-quality elite information, and recovering from stagnant search states is therefore desirable.

To address these issues, this study develops an Adaptive Reinforced Elite Gray Wolf Optimizer (AREGWO). Rather than relying on a single modification to the canonical GWO, AREGWO integrates several complementary mechanisms within the original leader-guided framework. Opposition-based initialization is introduced to improve initial search-space coverage. A nonlinear convergence factor dynamically regulates the transition from global exploration to local exploitation. Reinforced elite leadership increases the contribution of high-quality search information while retaining guidance from multiple leading wolves. Adaptive weak perturbation maintains population diversity during the early and intermediate stages of optimization, whereas a stagnation-aware partial restart selectively regenerates poorly performing individuals when population improvement ceases. Greedy elite inheritance is further employed to preserve high-quality solutions between successive iterations. Although the general ideas of opposition learning, nonlinear convergence control, elite guidance, perturbation, restart, and greedy selection have precedents in the metaheuristic literature, their specific formulation and coordination within AREGWO are designed around the search process of canonical GWO. The six mechanisms are selected to address different but complementary limitations: insufficient initial coverage, exploration–exploitation imbalance, weak utilization of elite information, loss of population diversity, search stagnation, and loss of high-quality solutions during population updating. Rather than acting as redundant operators, they are organized into a staged search architecture covering initialization, search regulation, population updating, diversity maintenance, stagnation recovery, and solution preservation. These mechanisms are designed to improve search diversity, local refinement, and stagnation recovery without substantially increasing the structural complexity of GWO. The individual operators are not claimed as independently new optimization principles. Rather, the methodological contribution of AREGWO lies in their coordinated integration at different stages of the GWO search process, including initialization, exploration–exploitation regulation, elite guidance, diversity maintenance, stagnation recovery, and solution preservation. Compared with recent GWO variants that primarily emphasize adaptive multi-strategy search [1], multi-population learning and mutation [8], or adaptive opposition-based search [9], AREGWO organizes complementary mechanisms across the successive stages of initialization, search regulation, leader-guided updating, diversity maintenance, stagnation recovery, and elite preservation within a unified GWO framework.

The proposed method is evaluated at three complementary levels. First, its numerical optimization capability and robustness are examined using the CEC2022 benchmark functions through comparisons with representative metaheuristic algorithms. Second, AREGWO is evaluated in synthetic 2D constrained robotic path-planning environments, where candidate solutions represent intermediate path-control points and the optimization objective jointly considers path length, directional variation, obstacle avoidance, and safety constraints. Third, AREGWO is coupled with TabM for football pass-quality prediction. In this application, AREGWO performs outer-level hyperparameter optimization, while TabM performs inner-level supervised regression using contextual, pressure-related, and historical cooperation features. Although robotic path planning and football prediction belong to different application domains, they were intentionally selected because they represent two fundamentally different optimization formulations. Robotic path planning evaluates a direct constrained search in a geometric solution space, whereas football prediction evaluates a model-level search in a hyperparameter space whose fitness is determined through neural-network training and validation; together, they test whether AREGWO can maintain effective search behavior across different decision representations and objective structures.

The main contributions of this study are summarized as follows.

(a) An AREGWO is developed through a structured redesign of the canonical GWO search process. Opposition-based initialization, nonlinear convergence control, and reinforced elite guidance enhance the initialization, exploration–exploitation regulation, and leader-guided updating stages, respectively, while adaptive weak perturbation, stagnation-aware partial restart, and greedy elite inheritance are coordinated to maintain population diversity, recover from stagnant states, and preserve high-quality solutions.
(b) An AREGWO-based path-planning framework is evaluated in synthetic 2D environments to extend the proposed optimizer from generic numerical optimization to constrained spatial optimization. The path-planning problem is formulated so that candidate solutions can be directly searched and updated by AREGWO under path-quality and obstacle-avoidance requirements.
(c) An AREGWO-driven neural tabular learning framework is developed for football pass-quality prediction. AREGWO performs outer-level hyperparameter optimization, while the neural tabular model performs inner-level supervised learning, thereby forming a unified bilevel optimization framework for data-driven prediction.

The remainder of this paper is organized as follows. Section 2 presents the biomimetic principles and mathematical formulation of AREGWO. Section 3 evaluates its numerical optimization performance using benchmark functions and comparative experiments. Section 4 formulates the robotic path-planning problem and presents the corresponding experimental validation. Section 5 introduces the football pass-quality prediction model, the AREGWO-based neural-model optimization framework, and the prediction and ablation experiments. Section 6 summarizes the principal findings.

## 2. Adaptive Reinforced Elite Gray Wolf Optimization

The original Gray Wolf Optimizer (GWO) is a swarm-intelligence algorithm inspired by the leadership hierarchy and cooperative hunting behavior of gray wolves, where the alpha, beta, and delta wolves guide the remaining population during the search process. Because of its simple structure and limited number of control parameters, GWO has been widely applied to continuous optimization and engineering problems. However, the canonical GWO may still suffer from premature convergence, insufficient population diversity, and an unstable balance between exploration and exploitation, especially when the search landscape is nonlinear or multimodal. Recent GWO variants have therefore introduced strategies such as dimension-learning hunting, adaptive search, elite inheritance, and hybrid mutation mechanisms to improve diversity and convergence stability.

In this study, AREGWO is developed as a compact population-based optimizer for general optimization tasks. Let *M_w_* denote the number of wolves, *D* denote the search dimension, and *τ*_max_ denote the maximum number of iterations. The position of the *m*-th wolf at iteration *τ* is denoted by

(1) $$\xi_{m}^{\left(\tau \right)}=[\xi_{m,1}^{\left(\tau \right)},\xi_{m,2}^{\left(\tau \right)},\ldots ,\xi_{m,K_{h}}^{\left(\tau \right)}],m=1,2,\ldots ,M_{w}$$

where $\xi_{m}^{\left(\tau \right)}$ represents the *m*th candidate solution and $\xi_{m,k}^{\left(\tau \right)}$ denotes its *k*th decision variable.

The lower and upper bounds of the hyperparameter search space are denoted by **b**^min^ and **b**^max^, respectively. The search range vector is defined as

(2) **ρ** = **b**^max^ − **b**^min^

where **ρ** denotes the element-wise range of the hyperparameter search space.

The first strategy of AREGWO is opposition-based initialization. For each randomly initialized wolf, an opposite candidate is generated as follows:

(3) $$\xi_{m}^{\mathrm{opp}}=b^{\min}+b^{\max}-\xi_{m}^{\left(0\right)}$$

where $\xi_{m}^{\left(0\right)}$ is the initial random position and $\xi_{m}^{\mathrm{opp}}$ is its opposite position. The original and opposite candidates are evaluated together, and the best *M_w_* candidates are selected to form the initial population. This strategy enlarges the initial search coverage and increases the probability of starting from promising regions.

For each candidate hyperparameter vector, the fitness function is defined as the validation error of the TabM model:

(4) $$F_{m}^{\left(\tau \right)}=J(\xi_{m}^{\left(\tau \right)})$$

where *J*(⋅) denotes the objective function, defined according to the specific optimization problem.

After the fitness evaluation, the population is sorted in ascending order of fitness. The best three wolves are denoted by

(5) $$\left(\xi_{\left[1\right]}^{\left(\tau \right)},\xi_{\left[2\right]}^{\left(\tau \right)},\xi_{\left[3\right]}^{\left(\tau \right)}\right)$$

where $\xi_{\left[1\right]}^{\left(\tau \right)}$, $\xi_{\left[2\right]}^{\left(\tau \right)}$, and $\xi_{\left[3\right]}^{\left(\tau \right)}$ correspond to the first-, second-, and third-best wolves in the current population, respectively. They play the roles of the alpha, beta, and delta wolves in the biomimetic hunting process.

Different from the linear convergence factor in the canonical GWO, AREGWO adopts a nonlinear cosine convergence factor:

(6) *χ*(*τ*) = 1 + cos(*πτ*/*τ*_max_)

where *χ*(*τ*) is the adaptive convergence factor and *τ* is the current iteration index. At the beginning of the search, *χ*(*τ*) is close to two, which encourages exploration. As the iteration proceeds, *χ*(*τ*) gradually decreases toward 0, which strengthens local exploitation around elite solutions.

For each leader *r* ∈ {1, 2, 3}, two random coefficient vectors are generated to simulate the encircling and hunting behaviors:

(7) $$m_{r}^{\left(\tau \right)}=2\chi (\tau )r_{r,1}^{\left(\tau \right)}-\chi (\tau ),n_{r}^{\left(\tau \right)}=2r_{r,2}^{\left(\tau \right)}$$

where $m_{r}^{\left(\tau \right)}$ and $n_{r}^{\left(\tau \right)}$ are coefficient vectors associated with the *r*-th leader, and $r_{r,1}^{\left(\tau \right)}$ and $r_{r,2}^{\left(\tau \right)}$ are random vectors uniformly distributed in [0, 1].

The candidate position guided by the *r*-th elite wolf is computed as

(8) $$z_{m,r}^{\left(\tau \right)}=\xi_{\left|r\right|}^{\left(\tau \right)}-m_{r}^{\left(\tau \right)}⊙\left|n_{r}^{\left(\tau \right)}⊙\xi_{\left|r\right|}^{\left(\tau \right)}-\xi_{m}^{\left(\tau \right)}\right|,r=1,2,3$$

where $z_{m,r}^{\left(\tau \right)}$ is the candidate position of the *m*-th wolf guided by the *r*-th elite wolf, and ⊙ denotes element-wise multiplication.

In the standard GWO, the three leader-guided positions are usually averaged. In contrast, AREGWO uses an alpha-dominant reinforced elite leadership strategy. The new candidate position is calculated as

(9) $${\tilde{\xi }}_{m}^{\left(\tau \right)}=0.5z_{m,1}^{\left(\tau \right)}+0.3z_{m,2}^{\left(\tau \right)}+0.2z_{m,3}^{\left(\tau \right)}$$

where ${\tilde{\xi }}_{m}^{\left(\tau \right)}$ is the newly generated candidate position. The fixed weights 0.5, 0.3, and 0.2 form a normalized hierarchical combination that follows the fitness ranking of the three leaders. Assigning 0.5 to the alpha wolf makes the best solution dominant without allowing a single leader to determine the update, while the remaining weights of 0.3 and 0.2 retain complementary guidance from the beta and delta wolves. The weights are therefore used as a simple rank-preserving elite allocation rather than as additional parameters requiring online adaptation.

To maintain diversity in the early search stage, AREGWO adds an adaptive weak perturbation to the newly generated candidate. The perturbation probability is defined as

(10) *μ*(*τ*) = 0.2(1 − *τ*/*τ*_max_)

where *μ*(*τ*) is the probability of perturbing each hyperparameter dimension. The perturbation is stronger in the early stage and gradually disappears in the later stage, which helps the optimizer explore a wider region at the beginning without disturbing final convergence.

The perturbed candidate is given by

(11) $${\tilde{\xi }}_{m}^{\left(\tau \right)}={\tilde{\xi }}_{m}^{\left(\tau \right)}+I_{m}^{\left(\tau \right)}⊙[0.05\chi (\tau )g_{m}^{\left(\tau \right)}⊙\rho ]$$

where ${\tilde{\xi }}_{m}^{\left(\tau \right)}$ is the perturbed candidate, $I_{m}^{\left(\tau \right)}$ is a binary mask sampled according to the probability *μ*(*τ*), and $g_{m}^{\left(\tau \right)}$ is a standard Gaussian random vector. The factor 0.05*χ*(*τ*)**ρ** controls the scale of the perturbation according to both the search range and the current exploration stage.

To avoid long-term stagnation, AREGWO further introduces a stagnation-aware partial restart mechanism. Let 𝒮*_stg_* denote the number of consecutive iterations without improvement. When 𝒮*_stg_* exceeds the stagnation threshold, the worst 20% of wolves are regenerated around the current best wolf:

(12) $${\hat{\xi }}_{m}^{\left(\tau \right)}=\xi_{\left[1\right]}^{\left(\tau \right)}+0.1h_{m}^{\left(\tau \right)}⊙\rho ,m\in W_{\mathrm{poor}}$$

where 𝒲*_poor_* denotes the subset of poorly performing wolves selected for restart, and $h_{m}^{\left(\tau \right)}$ is a standard Gaussian random vector. This mechanism does not restart the whole population. Instead, it preserves elite wolves and only refreshes poor candidates, thereby improving the population diversity while retaining the best search performance.

After perturbation or restart, boundary control is applied:

(13) $${\bar{\xi }}_{m}^{\left(\tau \right)}=\min[\max({\hat{\xi }}_{m}^{\left(\tau \right)},b^{\min}),b^{\max}]$$

where ${\bar{\xi }}_{m}^{\left(\tau \right)}$ is the feasible candidate after boundary correction. The operators min(·) and max(·) are applied element-wise.

Finally, AREGWO uses a greedy elite inheritance. Instead of directly replacing the old population with newly generated candidates, it merges the old and new populations and selects the best *M_W_* wolves:

(14) $$P^{\left(\tau +1\right)}={\mathrm{Best}}_{M_{W}}(P^{\left(\tau \right)}\cup {\bar{P}}^{\left(\tau \right)})$$

where 𝒫^(^*^τ^*^)^ is the old population, ${\bar{P}}^{\left(\tau \right)}$ is the corrected new population, and ${\mathrm{Best}}_{M_{W}}$ denotes the operation of selecting the *M_W_* candidates with the smallest fitness values. This inheritance strategy guarantees that high-quality solutions from the previous iteration are not lost during position updating.

The best fitness value obtained at each iteration is recorded to form the convergence curve. The detailed procedure of the proposed AREGWO is presented in Algorithm 1. As shown in Algorithm 1, AREGWO combines opposition-based initialization, nonlinear convergence control, reinforced elite leadership, adaptive weak perturbation, stagnation-aware partial restart, and greedy elite inheritance within a compact leader-guided search framework. These mechanisms act at different stages of the search process and serve complementary roles in population initialization, search regulation, diversity maintenance, stagnation recovery, and solution preservation.
**Algorithm 1.** AREGWOInput: Number of wolves *M_W_*; maximum iteration number *τ*_max_, lower bound **b**^min^; upper bound **b**^max^; search dimension *D*; fitness function 𝒥(⋅). Output: Best solution *ξ*_[1]_; best fitness 𝒥(*ξ*_[1]_).1.Compute the search range: **ρ** = **b**^max^ − **b**^min^.2.Randomly initialize *M_W_* wolves $\xi_{m}^{\left(0\right)}$, *m* = 1, 2, …, *M_W_*, within [**b**^min^,**b**^max^].3.Generate the opposite population: $\xi_{m}^{\mathrm{opp}}=b^{\min}+b^{\max}-\xi_{m}^{\left(0\right)}.$4.Evaluate all original and opposite wolves using 𝒥(⋅).5.Select the best *M_W_* wolves to form the initial population.6.Sort the population and determine the best three wolves $(\xi_{\left[1\right]}^{\left(\tau \right)},\xi_{\left[2\right]}^{\left(\tau \right)},\xi_{\left[3\right]}^{\left(\tau \right)}).$7.Set 𝒮*_stg_* = 0, and set the stagnation threshold as 𝒮_max_ = max(5, *round*(0.1*τ*_max_)).8.**for**  *τ* = 1 to *τ*_max_ do9.  Save the current population as 𝒫^(^*^τ^*^)^.10.  Calculate the adaptive convergence factor: *χ*(*τ*) = 1 + cos(*πτ*/*τ*_max_).11.  **for** *m* = 1 to *M_ω_* **do**12.    **for** *r* = 1 to 3 **do**13.      Generate coefficient vectors: $m_{r}^{\left(\tau \right)}=2\chi (\tau )r_{r,1}^{\left(\tau \right)}-\chi (\tau ),n_{r}^{\left(\tau \right)}=2r_{r,2}^{\left(\tau \right)}.$14.      Generate the elite-guided candidate: $z_{m,r}^{\left(\tau \right)}=\xi_{\left|r\right|}^{\left(\tau \right)}-m_{r}^{\left(\tau \right)}⊙\left|n_{r}^{\left(\tau \right)}⊙\xi_{\left|r\right|}^{\left(\tau \right)}-\xi_{m}^{\left(\tau \right)}\right|.$15.    **end for**16.    Generate the reinforced elite candidate: ${\tilde{\xi }}_{m}^{\left(\tau \right)}=0.5z_{m,1}^{\left(\tau \right)}+0.3z_{m,2}^{\left(\tau \right)}+0.2z_{m,3}^{\left(\tau \right)}.$17.  **end for**18.  Calculate the perturbation probability: *μ*(*τ*) = 0.2(1 − *τ*/*τ*_max_).19.  Generate binary perturbation mask $I_{m}^{\left(\tau \right)}$ according to *μ*(*τ*).20.  Apply adaptive weak perturbation: ${\tilde{\xi }}_{m}^{\left(\tau \right)}={\tilde{\xi }}_{m}^{\left(\tau \right)}+I_{m}^{\left(\tau \right)}⊙[0.05\chi (\tau )g_{m}^{\left(\tau \right)}⊙\rho ].$21.  **if** 𝒮*_stg_* ≥ 𝒮_max_22.   Select the worst 20% wolves as 𝒲*_poor_*.23.   Regenerate wolves in 𝒲*_poor_* around the best wolf: ${\hat{\xi }}_{m}^{\left(\tau \right)}=\xi_{\left[1\right]}^{\left(\tau \right)}+0.1h_{m}^{\left(\tau \right)}⊙\rho .$24.   Set 𝒮*_stg_* = 0.25.  **end if**26.  Apply boundary control: ${\bar{\xi }}_{m}^{\left(\tau \right)}=\min[\max({\hat{\xi }}_{m}^{\left(\tau \right)},b^{\min}),b^{\max}].$27.  Evaluate the corrected new population ${\bar{P}}^{\left(\tau \right)}$ using 𝒥(⋅).28.  Apply greedy elite inheritance: $P^{\left(\tau +1\right)}=\mathrm{Bes}t_{M_{W}}(P^{\left(\tau \right)}\cup {\bar{P}}^{\left(\tau \right)})$29.  Update $\left(\xi_{\left[1\right]}^{\left(\tau \right)},\xi_{\left[2\right]}^{\left(\tau \right)},\xi_{\left[3\right]}^{\left(\tau \right)}\right)$30.  **if** the best fitness is improved **then** set 𝒮*_stg_* = 0; **else** 𝒮*_stg_* = 𝒮*_stg_* + 1.31.  Record the current best fitness in the convergence curve.32.**end for**33.Return *ξ*_[1]_, 𝒥(*ξ*_[1]_).

## 3. Performance Evaluation of AREGWO

### 3.1. CEC2022 Benchmark Evaluation

To comprehensively evaluate the optimization performance and robustness of the proposed Adaptive Reinforced Elite Gray Wolf Optimizer (AREGWO), experiments were conducted on the CEC2022 benchmark suite. The comparison set was expanded to include both classical population-based optimizers and more recent or stronger optimization methods, thereby enabling AREGWO to be assessed against its canonical predecessor, established swarm-intelligence algorithms, recent GWO variants, and a competitive differential-evolution-based optimizer.

GWO was retained as the canonical baseline because AREGWO was developed from the original Gray Wolf Optimizer. It uses a hierarchical leader-guided search mechanism based on the alpha, beta, and delta wolves [5].

PSO was selected as a classical swarm-intelligence optimizer. It updates candidate solutions through velocity adjustment based on individual and global experience [28].

DBO was included as a recent bio-inspired optimizer. It simulates multiple dung beetle behaviors to balance global exploration and local exploitation [29].

WOA was selected as a representative nature-inspired metaheuristic. Its search process combines encircling, spiral updating, and random exploration [30].

IAGWO was included as a recent GWO variant. It introduces adaptive and multi-strategy search mechanisms to improve the exploration–exploitation capability of the original GWO [1].

AOGWO was selected as another recent GWO variant. It combines opposition-based learning with adaptive search regulation to enhance the population diversity and convergence behavior [9].

L-SHADE was included as a strong differential-evolution-based optimizer. It combines success-history parameter adaptation with linear population-size reduction [31].

DIO was selected as a recent nature-inspired optimizer. It models cooperative hunting and adaptive interaction among dholes to guide population evolution [32,33].

Recent studies have additionally demonstrated the continuing use of biological optimization in data-driven tasks, such as Satin Bowerbird Optimization for deep-feature selection in transformer-based image classification [34]. These developments further motivate the inclusion of both recent bio-inspired methods and established strong optimizers when evaluating a newly proposed metaheuristic.

For all algorithms, the CEC2022 problem dimension was set to 20, the initial population size to 30, the maximum number of iterations to 1000, and the number of independent runs to 30. The search domain of each benchmark function was set according to the standard CEC2022 definition, with all decision variables bounded within [−100, 100]. Identical benchmark-function definitions and boundary-handling conditions were used for all methods. The algorithm-specific parameter settings are summarized in Table 1.

The stopping condition was defined by the maximum number of iterations rather than by an identical objective-function evaluation budget. Therefore, the exact number of objective-function evaluations was not strictly identical among all algorithms because their internal evaluation structures differ. In particular, AREGWO evaluates both the original and opposite populations during opposition-based initialization, resulting in an additional 2M objective-function evaluations at the initial stage. Other algorithms retain their original algorithm-specific evaluation procedures. Accordingly, the comparison should be interpreted as being conducted under identical population sizes and iteration settings, rather than under a strictly identical function-evaluation budget. The additional computational effort introduced by algorithm-specific mechanisms is reflected in the reported MeanTime.

All experiments were executed in MATLAB R2021b on a Windows 10 workstation equipped with an Intel Core i7-12700 CPU and 32 GB RAM.

**Figure 1:**
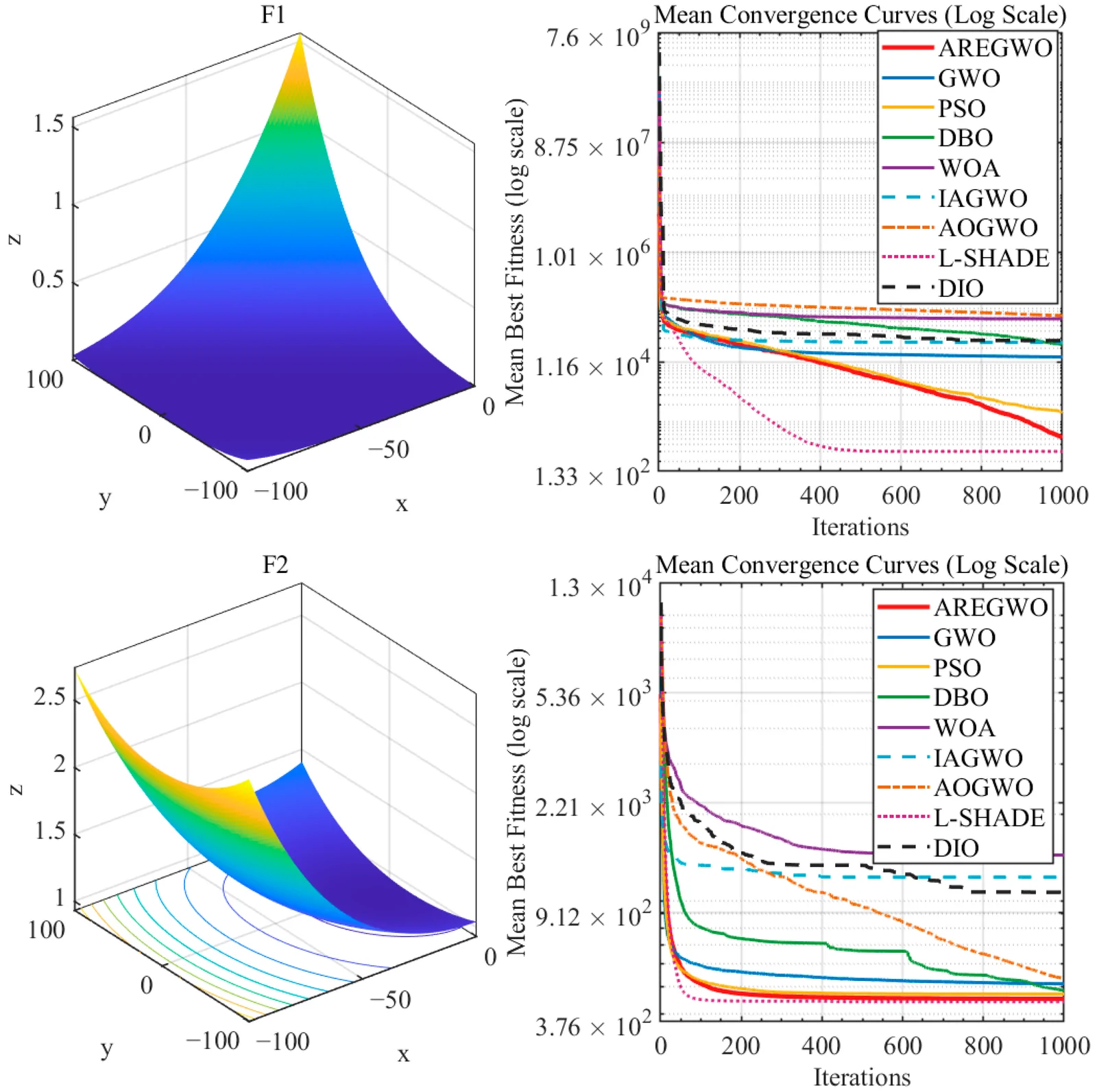

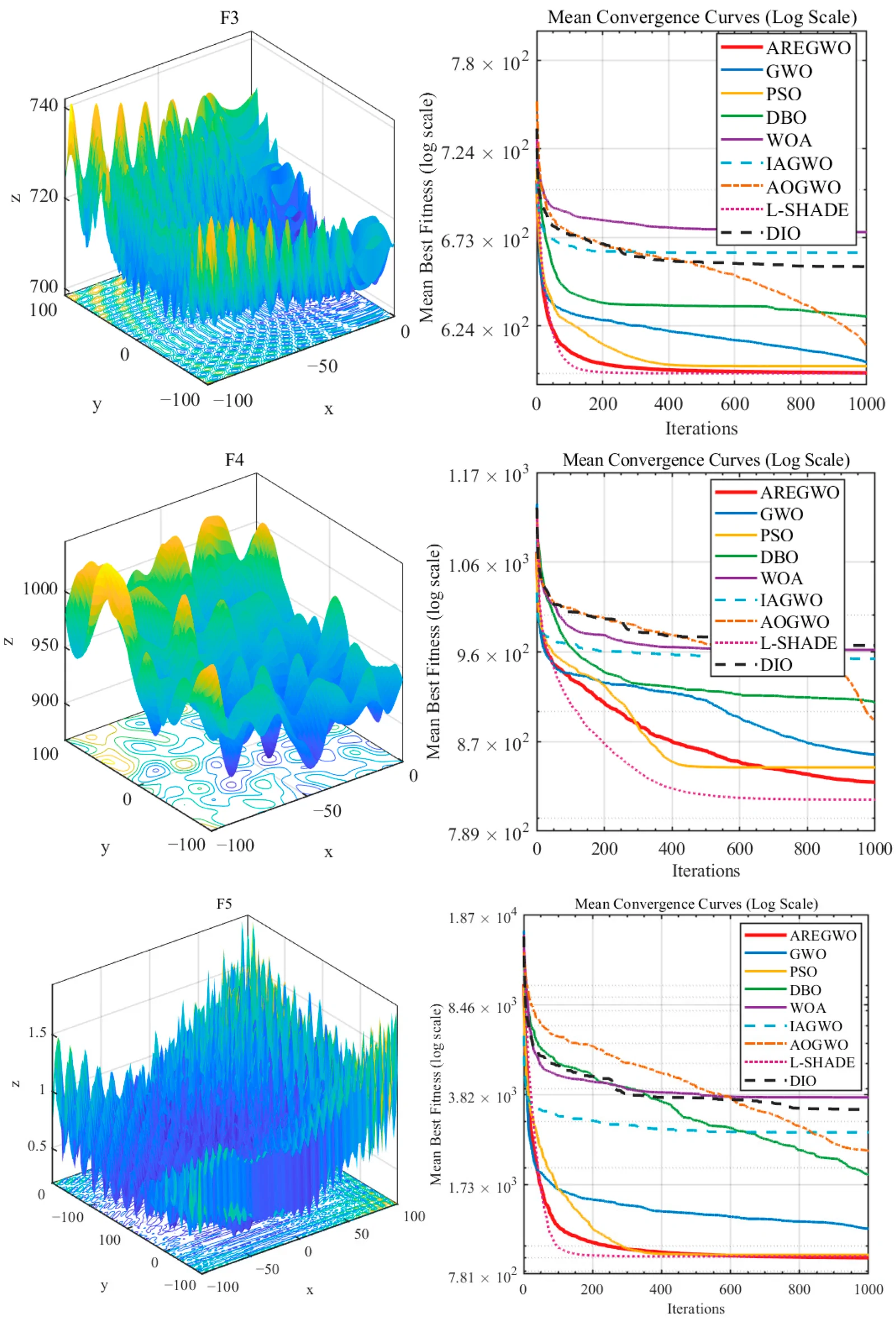

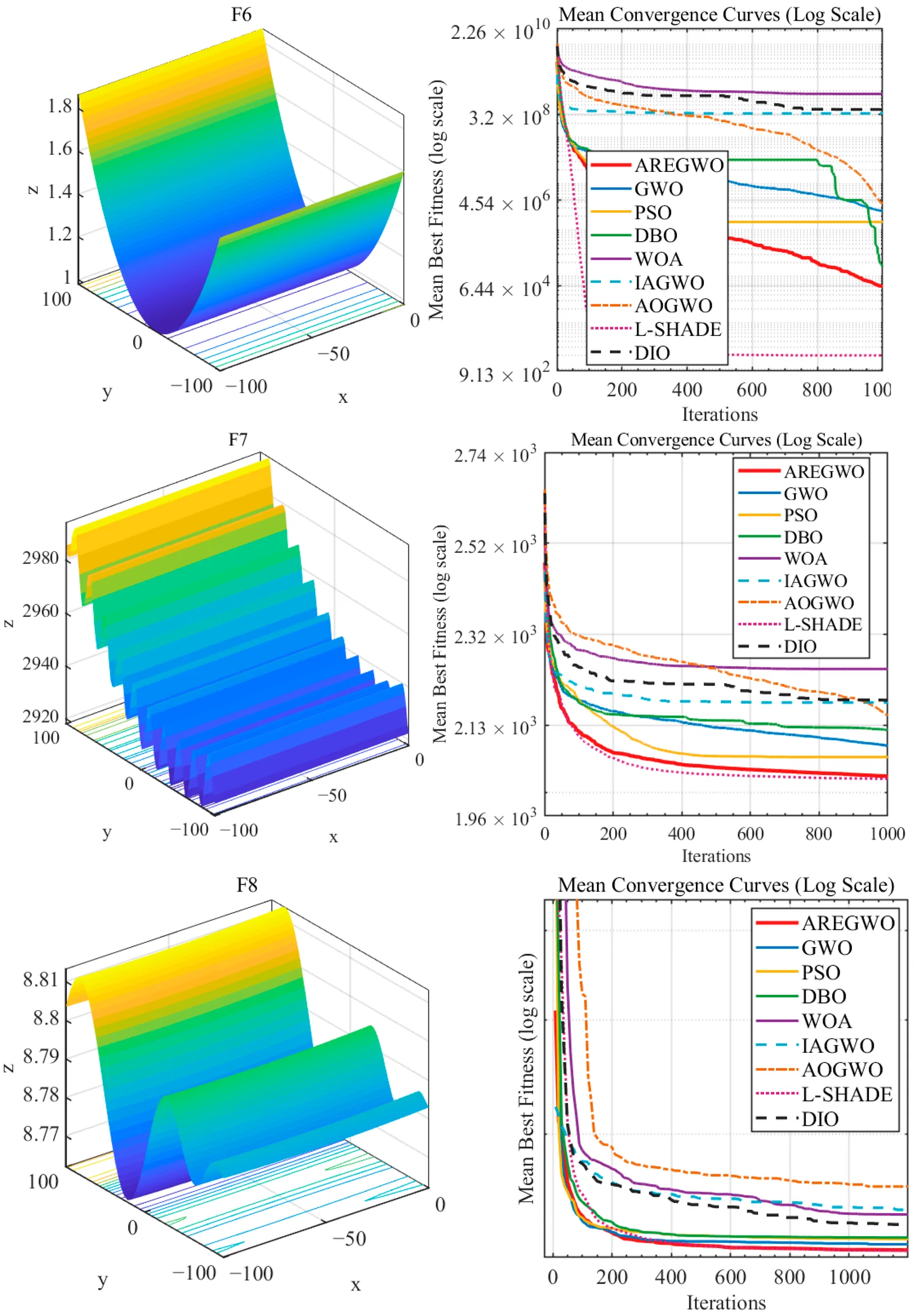

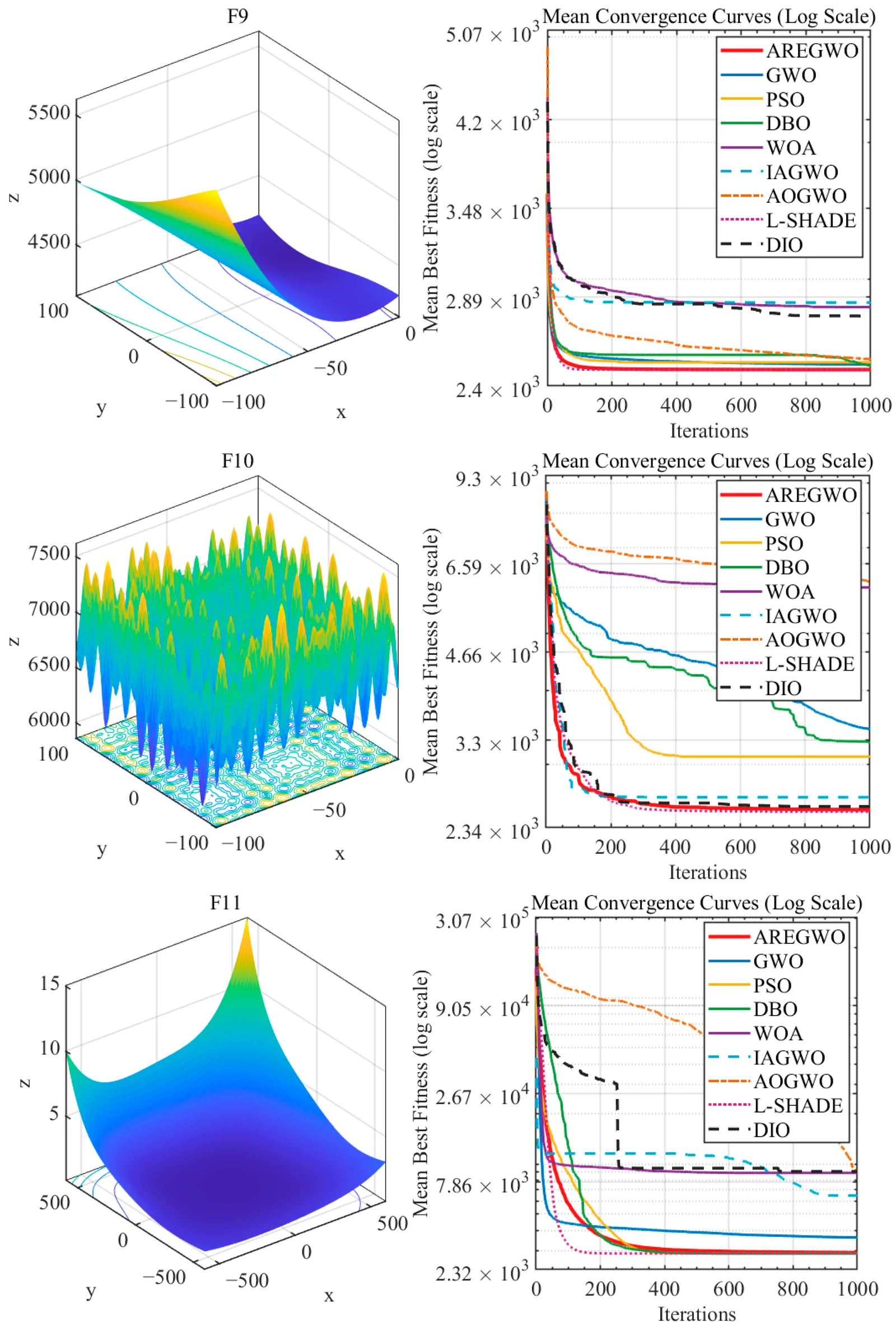

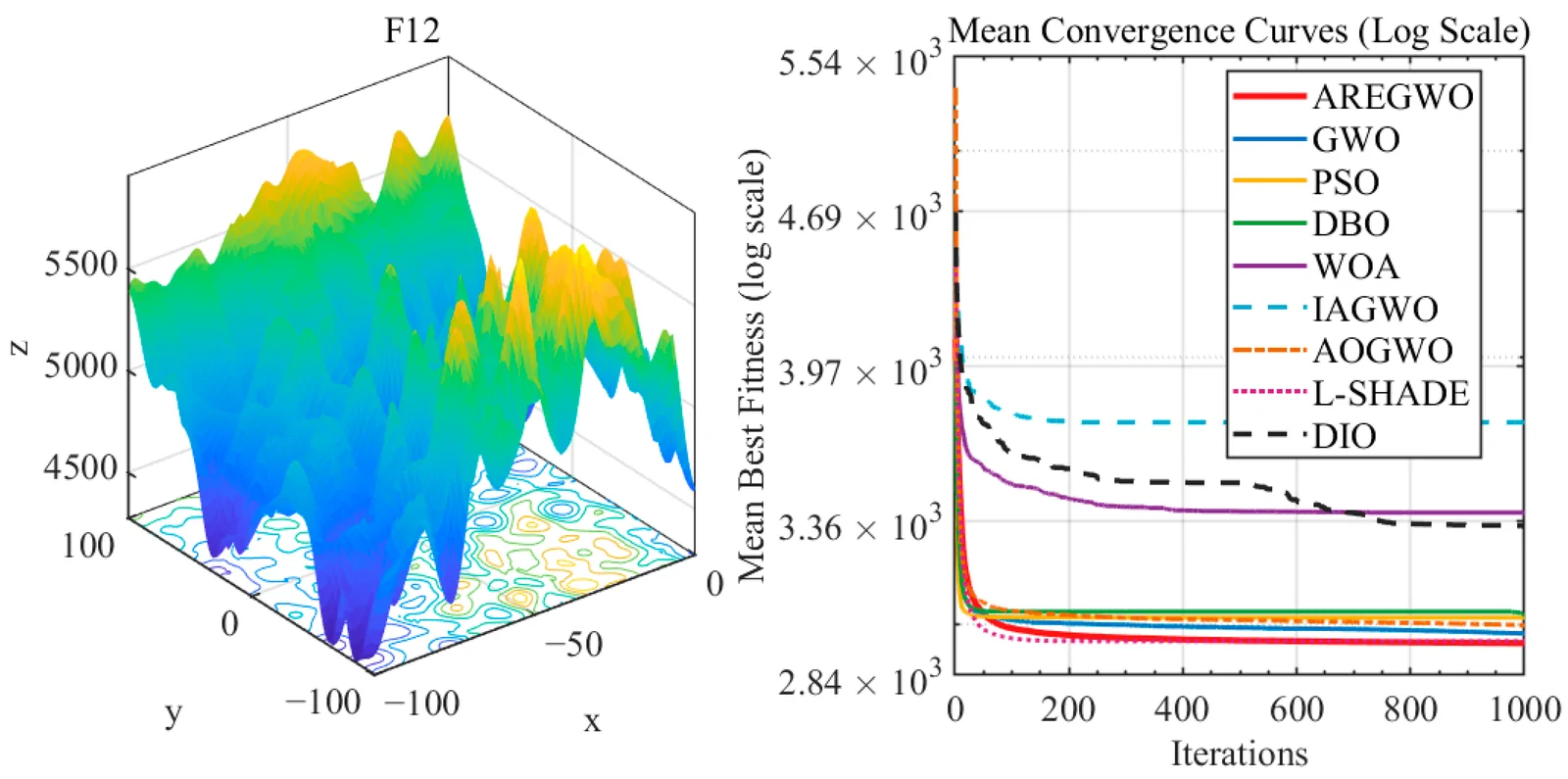
Convergence-curve comparison of AREGWO and the comparison algorithms on the CEC2022 benchmark functions.

**Figure 2:**
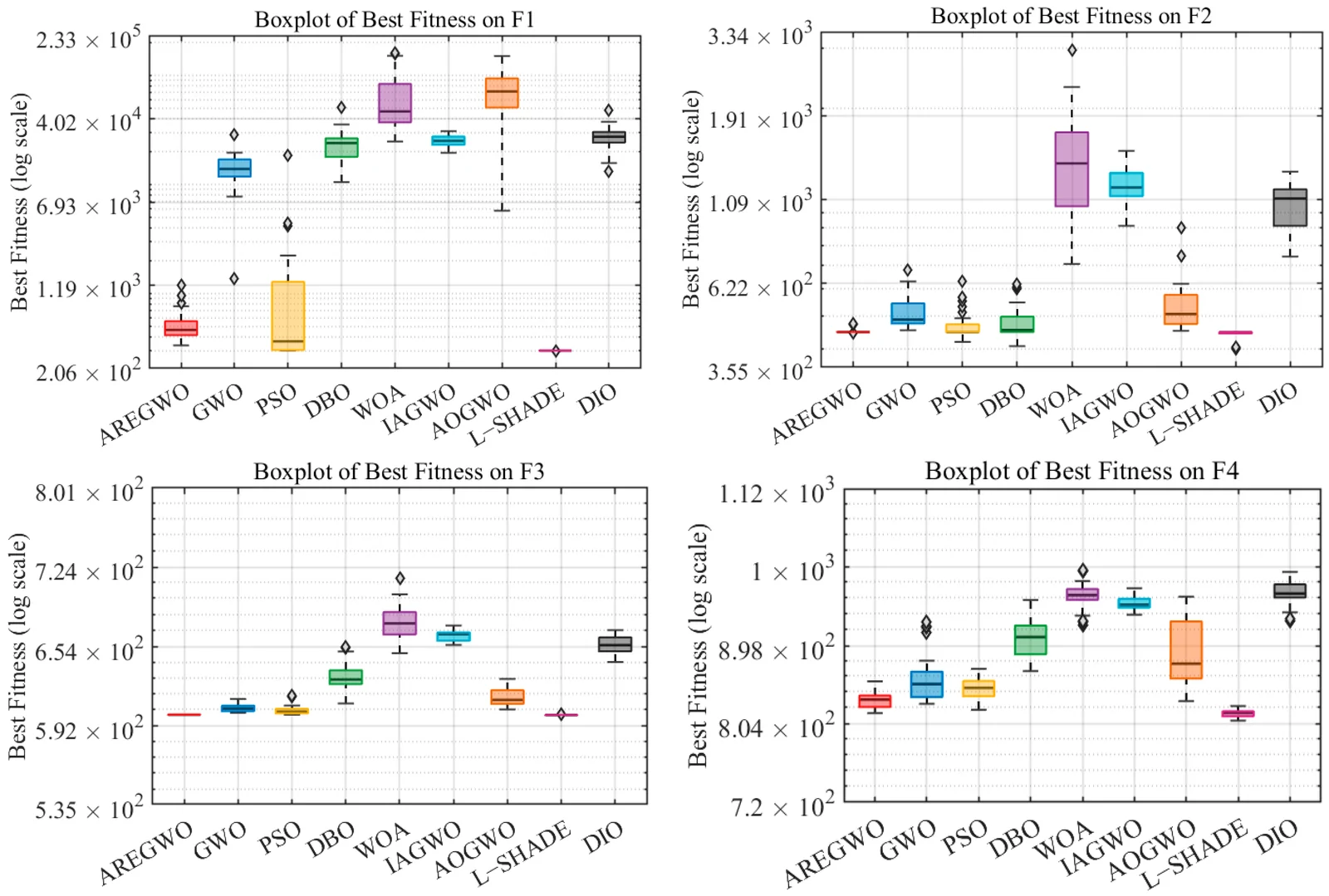

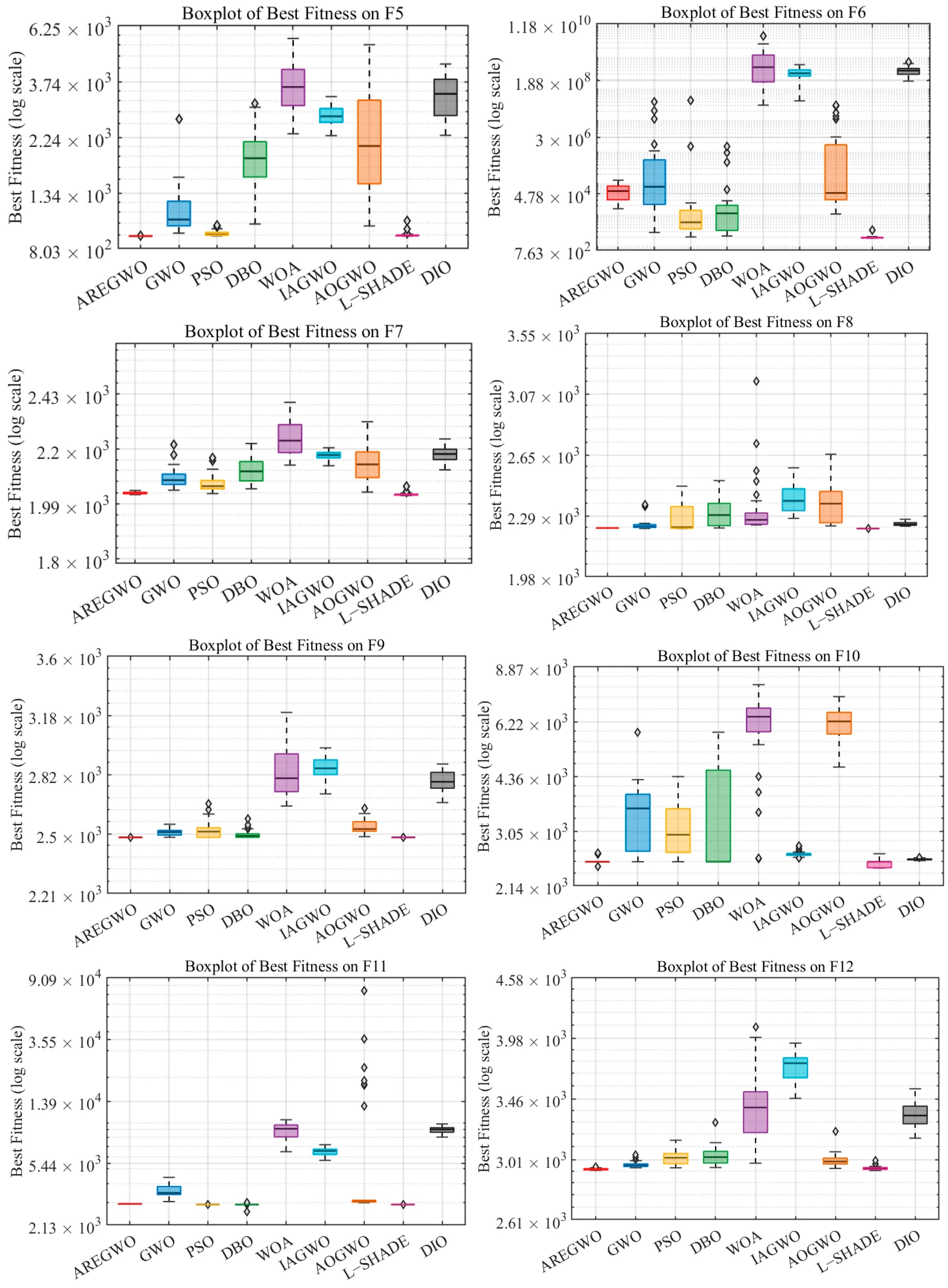
Boxplot comparison of final fitness values obtained by different algorithms over 30 independent runs.

All algorithms were evaluated under a consistent experimental framework. The initial population size was set to 30, the maximum number of iterations to 1000, and the search dimension to 20, while identical benchmark-function definitions and boundary conditions were used. Algorithm-specific internal adaptation mechanisms were retained. Each CEC2022 function was independently executed 30 times to reduce the influence of stochastic variation. Performance was evaluated using Mean, Std., Best, Worst, and MeanTime. Mean represents the average final fitness over 30 runs, Std. reflects run-to-run stability, Best and Worst denote the best and worst final fitness values, respectively, and MeanTime represents the average computational time. The convergence behavior, distribution of final fitness values, numerical results, and statistical comparisons are presented in Figure 1, Figure 2, Table 2, and Table 3, respectively.

As shown in Figure 1, the convergence curves reveal clear differences in the search behavior of the nine algorithms across the CEC2022 benchmark functions. Most algorithms achieve a rapid fitness reduction during the early iterations, whereas their middle- and late-stage behaviors differ substantially. AREGWO generally maintains fast initial convergence and continues to improve on several functions, achieving competitive or near-best final fitness on F2, F3, F5, F7–F9, F11, and F12. On F6, AREGWO continues to decrease markedly in the later search stage after several competing algorithms have already stagnated, indicating a stronger capability for maintaining search progress in difficult landscapes. L-SHADE exhibits particularly strong convergence on several functions, especially F1, F4, F6, and F7, and frequently reaches the lowest or nearly lowest final fitness among all methods. In comparison, the standard GWO and PSO show competitive behavior on some functions but tend to exhibit slower late-stage improvement, while DBO and WOA generally converge at higher fitness levels on a number of functions. The recent GWO variants IAGWO and AOGWO also show effective search behavior on several benchmarks, but their performance varies more noticeably across different landscapes. DIO demonstrates competitive convergence on several functions, although it does not consistently outperform AREGWO. The expanded comparison indicates that AREGWO does not dominate every benchmark function, but it maintains consistently competitive convergence across diverse problem landscapes and shows a favorable balance between convergence speed, final solution quality, and continued search capability.

The robustness of the nine algorithms over 30 independent runs is further illustrated by the boxplots in Figure 2. The distributions reveal substantial differences in both optimization accuracy and run-to-run variability across the CEC2022 functions. AREGWO generally exhibits relatively low median fitness and compact distributions on several functions, particularly F2, F3, F5, F7–F9, F11, and F12, indicating stable and repeatable search performance across different problem landscapes. L-SHADE shows the strongest distributional performance on a number of functions, with particularly narrow boxes and low median values on F1, F4, F6–F10, and F11, demonstrating high robustness under repeated runs. In comparison, the standard GWO and PSO achieve relatively compact distributions on some functions but show larger dispersion or higher median fitness on others. DBO and WOA exhibit noticeably wider interquartile ranges and more dispersed outliers on several benchmarks, indicating greater sensitivity to stochastic variation. The recent GWO variants IAGWO and AOGWO also show competitive robustness on selected functions, although their median fitness and dispersion vary more substantially across different landscapes. DIO presents relatively stable distributions on several functions, but its final fitness is generally higher than those of the best-performing methods. Overall, the boxplot results indicate that AREGWO provides a favorable combination of solution quality and repeatability across the benchmark suite, although L-SHADE achieves superior robustness on several individual functions. This observation is consistent with the convergence results in Figure 1 and further demonstrates the competitive overall performance of AREGWO under repeated stochastic runs.

The numerical results in Table 2 further quantify the convergence and robustness characteristics observed in Figure 1 and Figure 2. In terms of mean final fitness, AREGWO achieves the best or near-best performance on a substantial proportion of the CEC2022 functions. It remains highly competitive on F2, F3, F5, F7–F9, F11, and F12, while on F1 and F6, it ranks behind L-SHADE but substantially outperforms most of the remaining comparison algorithms. The advantage over the canonical GWO is particularly pronounced on difficult functions such as F1 and F6. On F1, AREGWO obtains a mean fitness of 5.32 × 10^2^, compared with 1.40 × 10^4^ for GWO, while on F6, it achieves 1.00 × 10^4^, substantially lower than the 2.60 × 10^6^ obtained by GWO and the considerably larger values produced by several other population-based methods. L-SHADE nevertheless shows superior numerical accuracy on several functions, particularly F1, F4, F6, F7, F8, and F10, demonstrating the competitiveness of this strong evolutionary baseline. These results indicate that AREGWO provides consistently competitive optimization accuracy across heterogeneous search landscapes, although its relative advantage varies with problem characteristics.

In terms of run-to-run stability, AREGWO exhibits relatively small standard deviations on several functions, with particularly stable performance on F2, F5, F11, and F12. However, L-SHADE achieves smaller dispersion on a number of other functions, and some competing methods also show strong stability on specific landscapes. The Best and Worst values exhibit a similar pattern: AREGWO generally maintains favorable solution quality across repeated runs, but no single algorithm dominates all benchmark functions. Regarding computational efficiency, the additional search operations in AREGWO introduce a moderate computational overhead compared with several simpler swarm optimizers, while its MeanTime remains substantially lower than that of L-SHADE across the tested functions. Overall, Table 2 shows that AREGWO achieves a favorable balance among optimization accuracy, robustness, and computational cost. Its consistent performance across the CEC2022 suite supports its use as the hyperparameter optimizer in the subsequent AREGWO-TabM framework.

Based on Table 3, the statistical analysis confirms significant performance differences among the nine algorithms across the CEC2022 benchmark suite. The Friedman test gives a chi-square statistic of 74.600 with a *p*-value of 5.93 × 10^−13^. In terms of mean rank, L-SHADE ranks first with 1.250, followed by AREGWO with 2.000, while the remaining algorithms obtain higher mean ranks. These results indicate that AREGWO achieves the second-best overall performance among the nine compared methods, although L-SHADE remains the strongest competitor across the benchmark suite.

To further examine pairwise differences, two-sided Wilcoxon signed-rank tests were performed using the 12 benchmark functions as paired statistical units, with AREGWO taken as the reference algorithm. Because eight pairwise comparisons were conducted, the resulting *p*-values were adjusted using the Holm–Bonferroni procedure. After correction, AREGWO remains significantly better than GWO, PSO, DBO, WOA, IAGWO, AOGWO, and DIO, whereas L-SHADE remains significantly better than AREGWO. The W/T/L results are consistent with these findings: AREGWO achieves lower mean fitness than several competitors on all 12 functions and outperforms PSO and DBO on 11 of the 12 functions, while it performs worse than L-SHADE on most functions.

The rank-biserial correlations further quantify the magnitude and direction of the pairwise differences. Positive effect sizes are obtained for the comparisons with the seven algorithms outperformed by AREGWO, whereas the negative effect size against L-SHADE confirms the overall advantage of L-SHADE. Overall, the statistical results are consistent with the numerical and convergence analyses and support the strong competitiveness of AREGWO across heterogeneous CEC2022 landscapes while also indicating that its performance advantage is problem-dependent rather than universal.

Collectively, the CEC2022 results demonstrate that AREGWO achieves strong and consistently competitive optimization performance across diverse benchmark landscapes. The convergence curves show that AREGWO maintains effective search progress over different optimization stages, while the boxplots indicate generally stable performance over 30 independent runs. The numerical results further show that AREGWO attains competitive Mean, Best, and Std. values on a broad range of functions, although L-SHADE exhibits superior performance on several benchmarks. The Friedman ranking places AREGWO second among the nine evaluated algorithms, and the Wilcoxon signed-rank test shows that AREGWO significantly outperforms seven of the eight comparison methods, with L-SHADE remaining the strongest competitor. Overall, these results indicate that AREGWO provides a favorable balance among optimization accuracy, robustness, and computational efficiency, supporting its use in the subsequent application studies.

### 3.2. Component Ablation Analysis

To investigate the contribution of the main components of AREGWO, a leave-one-out ablation study was conducted on three representative CEC2022 benchmark functions: F1, F6, and F12. For each ablation model, only the specified component was removed or replaced by its corresponding basic GWO operation, while the remaining structure and parameter settings were kept unchanged. The six ablation models are defined as follows.

w/o OBI. The opposition-based competitive initialization is removed, and the initial population is generated only by uniform random sampling within the search bounds. All subsequent AREGWO search mechanisms remain unchanged.

w/o NCF. The nonlinear convergence control is removed and replaced by the linear convergence factor of the original GWO. This variant is used to evaluate the effect of adaptive exploration–exploitation regulation.

w/o REL. The reinforced elite leadership mechanism is removed, and the three leading wolves are combined using the equal-weight guidance strategy of the original GWO. This variant evaluates the contribution of enhanced elite information utilization.

w/o PERT. The adaptive weak perturbation mechanism is disabled, while the remaining population-update operations are retained. This variant is used to examine the role of additional stochastic perturbation in maintaining search diversity.

w/o RESTART. The stagnation-triggered reconstruction mechanism is removed. The population therefore continues to evolve without an explicit restart when prolonged stagnation is detected.

w/o GREEDY. The greedy elite inheritance mechanism is removed, and the newly generated population directly replaces the previous population after boundary correction. This variant is used to evaluate the effect of retaining favorable population updates during successive iterations.

Each ablation variant was independently executed 30 times on each selected function. The corresponding results are presented in Table 4. On F1, removing OBI, NCF, REL, PERT, or RESTART increases the mean fitness from 5.26 × 10^2^ to 5.44 × 10^2^–6.44 × 10^2^, indicating that these mechanisms generally contribute positively to the optimization process. Among them, removing NCF produces the largest deterioration. In contrast, w/o GREEDY obtains a lower mean value of 3.98 × 10^2^, indicating that greedy selection is not uniformly advantageous for every search landscape.

On F6, the complete AREGWO achieves the best mean fitness of 4.82 × 10^4^. Removing any component leads to performance degradation, and the effects of RESTART and GREEDY are particularly evident. The mean fitness increases to 6.88 × 10^4^ without RESTART and further to 5.57 × 10^5^ without GREEDY. The substantially increased standard deviation without GREEDY also indicates reduced robustness. For F12, the differences among most variants are relatively small, whereas removing GREEDY slightly worsens both the mean fitness and stability.

The effect of reinforced elite guidance is also visible in the ablation results. Replacing the proposed 0.5/0.3/0.2 hierarchical weighting with the equal-weight guidance of standard GWO increases the mean fitness from 5.26 × 10^2^ to 5.44 × 10^2^ on F1 and from 4.82 × 10^4^ to 6.16 × 10^4^ on F6, while the difference in F12 is relatively small. This result indicates that emphasizing the alpha wolf while retaining beta- and delta-guided information can improve the search on some landscapes, although the benefit remains problem-dependent.

The ablation results indicate that the components of AREGWO play different and complementary roles. OBI, NCF, REL, PERT, and RESTART generally improve optimization performance on F1 and F6, whereas GREEDY exhibits stronger problem dependence. The complete AREGWO therefore does not rely on a single dominant mechanism; instead, its overall performance results from the coordinated interaction of initialization, convergence regulation, elite guidance, diversity maintenance, stagnation recovery, and selection mechanisms.

## 4. Robotic Path Planning

### 4.1. Robotic Path Planning Model

To evaluate the applicability of AREGWO to constrained engineering optimization, a two-dimensional mobile robot path-planning problem is considered. The robot moves from a prescribed start point to a goal point in an environment containing obstacles. A candidate solution is represented by several intermediate control points, which determine the overall route between the start and goal positions. In the implemented model, each control point contains two normalized decision variables, and the complete optimization vector is defined as

(15) $$u=\left[u_{1},u_{2},\ldots ,u_{2K}\right],0\leq u_{i}\leq 1$$

where **u** is the path-planning decision vector, *K* is the number of intermediate control points, and *u_i_* is the *i*-th normalized decision variable. Therefore, the dimension of the optimization problem is 2*K*. In the present implementation, four control points are used, corresponding to an eight-dimensional search space.

To maintain an approximately ordered distribution of the control points from the start to the goal, each point is assigned a longitudinal position along the start-goal direction together with an adjustable lateral displacement. The k-th requested control point can be expressed as

(16) **P***_k_* = **P***_s_* + *a_k_***e**_1_ + *b_k_***e**_2_

where **P***_s_* is the start point, **e**_1_ is the unit vector from the start point to the goal point, **e**_2_ is the corresponding perpendicular unit vector, *a_k_* is the longitudinal position of the *k*-th control point, and *b_k_* is its lateral displacement. The longitudinal coordinate is restricted to an ordered interval, while the lateral coordinate is mapped to the valid map boundary. This design allows the optimizer to adjust the route shape while reducing disordered control-point distributions.

Obstacle avoidance is evaluated using a Euclidean distance field generated from the occupancy map. The minimum allowable clearance is defined as

(17) *d*_min_ = *r_bot_* + *d_grid_*

where *r_bot_* is the robot radius and *d_grid_* is the additional conservative margin associated with the grid resolution. A control point or path segment is regarded as feasible only when its distance from the obstacle region is not smaller than *d*_min_. In the program, the complete path segment is sampled during collision checking rather than evaluating only its endpoints. Inthe experiments, *r_bot_* = 0.35 m. With a grid resolution of 2 cells/m, the grid spacing is 0.5 m, and the conservative grid-dependent margin is set to the diagonal length of one grid cell, resulting in *d*_min_ = 1.057 m.

If a generated control point is located in an unsafe or inaccessible region, it is projected to the nearest connected safe node. For two adjacent control points, a straight connection is retained when the segment satisfies the clearance requirement; otherwise, a local safe roadmap is used to construct an obstacle-avoiding connection. After all path segments are generated, redundant turning points are removed when the direct connection remains collision-free. This procedure preserves the required control points while reducing unnecessary detours.

The objective function evaluates the actual generated route and simultaneously considers path length, directional variation, and the correction required for infeasible control points:

(18) *J_path_* = *L_path_* + *ω*_1_*T_path_* + *ω*_2_*D_rep_*

where *J_path_* is the path-planning objective value, *L_path_* is the total traveled distance, *T_path_* is the sum of squared turning angles along the route, *D_rep_* is the accumulated displacement introduced by control-point repair, and *ω*_1_ = 1.5 and *ω*_2_ = 10 are the corresponding weighting coefficients. It should be noted that the turning and repair terms in Equation (18) are introduced to guide the continuous population-based search and constraint handling. In particular, *D_rep_* represents an internal control-point repair cost and has no direct counterpart in the A search. Therefore, the composite search fitness is not used for direct cross-algorithm performance comparison. For all methods, the final route is evaluated using the actual path length under the same collision and safety-clearance constraints.

To provide a more comprehensive evaluation of path-planning performance, four metrics are considered, including path length, computation time, success rate, and minimum safety distance. For *N_r_* independent runs, these metrics are defined as

(19) $$\left\{\begin{array}{l} {\bar{L}}_{\mathrm{path}}=\frac{1}{N_{s}}\sum_{r:s_{r}=1}\sum_{i=1}^{N_{p}^{\left(r\right)}-1}{\left\|P_{i+1}^{\left(r\right)}-P_{i}^{\left(r\right)}\right\|}_{2}\\ \bar{T}=\frac{1}{N_{r}}\sum_{r=1}^{N_{r}}T^{\left(r\right)}\\ \mathrm{SR}=\frac{1}{N_{r}}\sum_{r=1}^{N_{r}}s_{r}\times 100\%\\ {\bar{d}}_{\min}=\frac{1}{N_{s}}\sum_{r:s_{r}=1}\min_{p\in P(r),q\in \partial O}{\left\|p-q\right\|}_{2} \end{array}\right.$$

where *N_r_* is the total number of independent runs, *s_r_* ∈ {0, 1} is the success indicator of the *r*-th run, and $N_{s}=\sum_{r=1}^{N_{r}}s_{r}$ is the number of successful runs. A run is regarded as successful when the generated route connects the start and goal while satisfying the collision and prescribed safety-clearance constraints. $N_{p}^{\left(r\right)}$ denotes the number of points on the final route of the *r*-th successful run, and $P_{i}^{\left(r\right)}$ is its *i*-th route point. *T*^(^*^r^*^)^ denotes the computation time of the *r*-th run. 𝒫^(^*^r^*^)^ represents the final route, while ∂𝒪 denotes the boundaries of all obstacles. Accordingly, ${\bar{L}}_{\mathrm{path}}$, $\bar{T}$, *SR*, and ${\bar{d}}_{\min}$ represent the mean path length, mean computation time, success rate, and mean minimum safety distance, respectively.

The resulting optimization problem is finally written as

(20) $$u*=\arg\min_{u\in {\left[0,1\right]}^{2K}}J_{\mathrm{path}}(u)$$

where u* is the optimal path control vector obtained by AREGWO.

### 4.2. Comparative Evaluation of Robotic Path Planning

To evaluate the path-planning performance and environmental adaptability of AREGWO, five representative methods were selected for comparison, including GWO, PSO, DBO, WOA, and A search. Nature-inspired optimization has also been applied to related discrete routing problems, including traveling-salesman and tourism-routing tasks [35]. For the population-based algorithms, the population size and maximum number of iterations were set to 40 and 150, respectively, and four intermediate control points were optimized for each candidate path. Six independently generated random maps with different obstacle distributions and start–goal configurations were considered to provide a broader evaluation under varying spatial conditions. The same map, start and goal positions, and safety-clearance requirements were used for all algorithms within each environment. Each stochastic optimization algorithm was independently executed 30 times on each map. The four metrics defined in Equation (19), namely path length, computation time, success rate, and minimum safety distance, were jointly used for quantitative evaluation.

All test environments were generated on 100 m × 100 m maps represented by a 201 × 201 occupancy grid with a resolution of 2 cells/m. Rectangular, diamond-shaped, circular, and triangular obstacles were generated using a best-candidate placement procedure, and the obstacle number was varied among the six maps to obtain different environmental densities. The generated maps were fixed before optimization and reused for all algorithms.

For a consistent comparison, the routes returned by all methods were evaluated using the same feasibility and collision-checking protocol. Safety-preserving smoothing was accepted only when the shortened segment remained collision-free and satisfied the prescribed clearance. The complete route was subsequently rechecked using the same distance field and segment-sampling procedure, and routes failing this final check were counted as unsuccessful runs. The final cross-algorithm comparison was therefore based on the common route-level metrics defined in Equation (19), rather than on the algorithm-specific internal fitness values.

**Figure 3:**
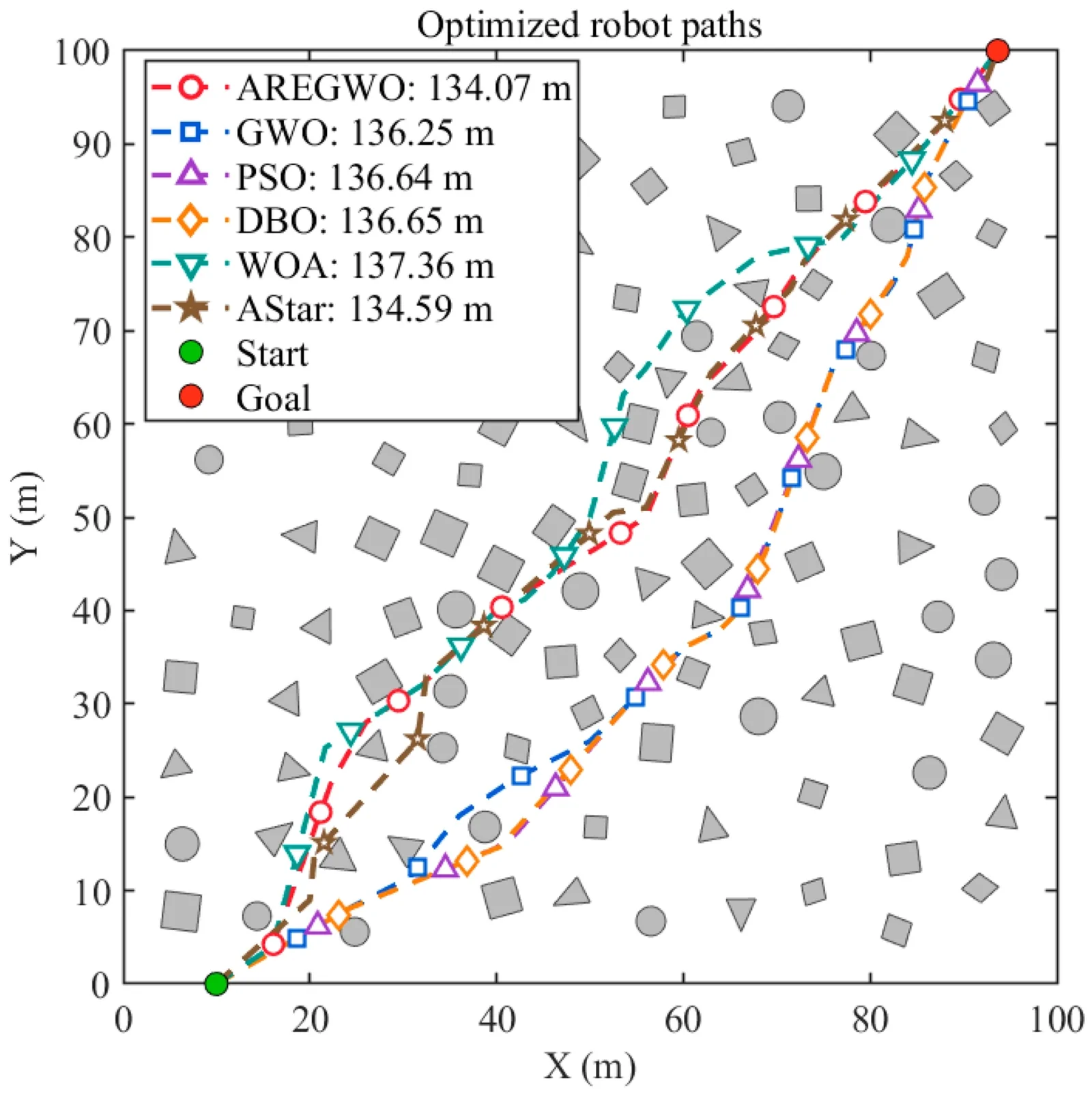
Comparative path-planning results on Random Map 1.

**Figure 4:**
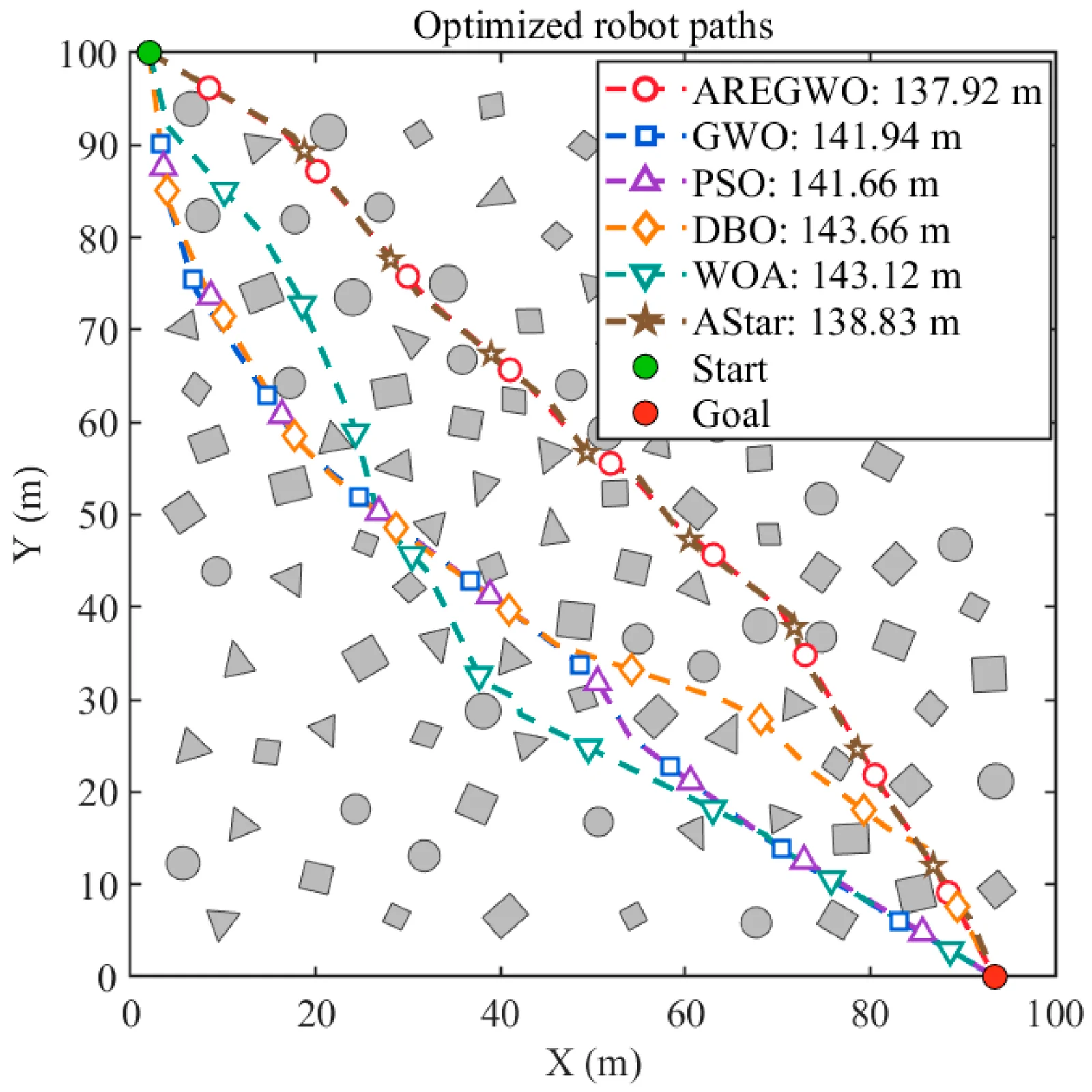
Comparative path-planning results on Random Map 2.

**Figure 5:**
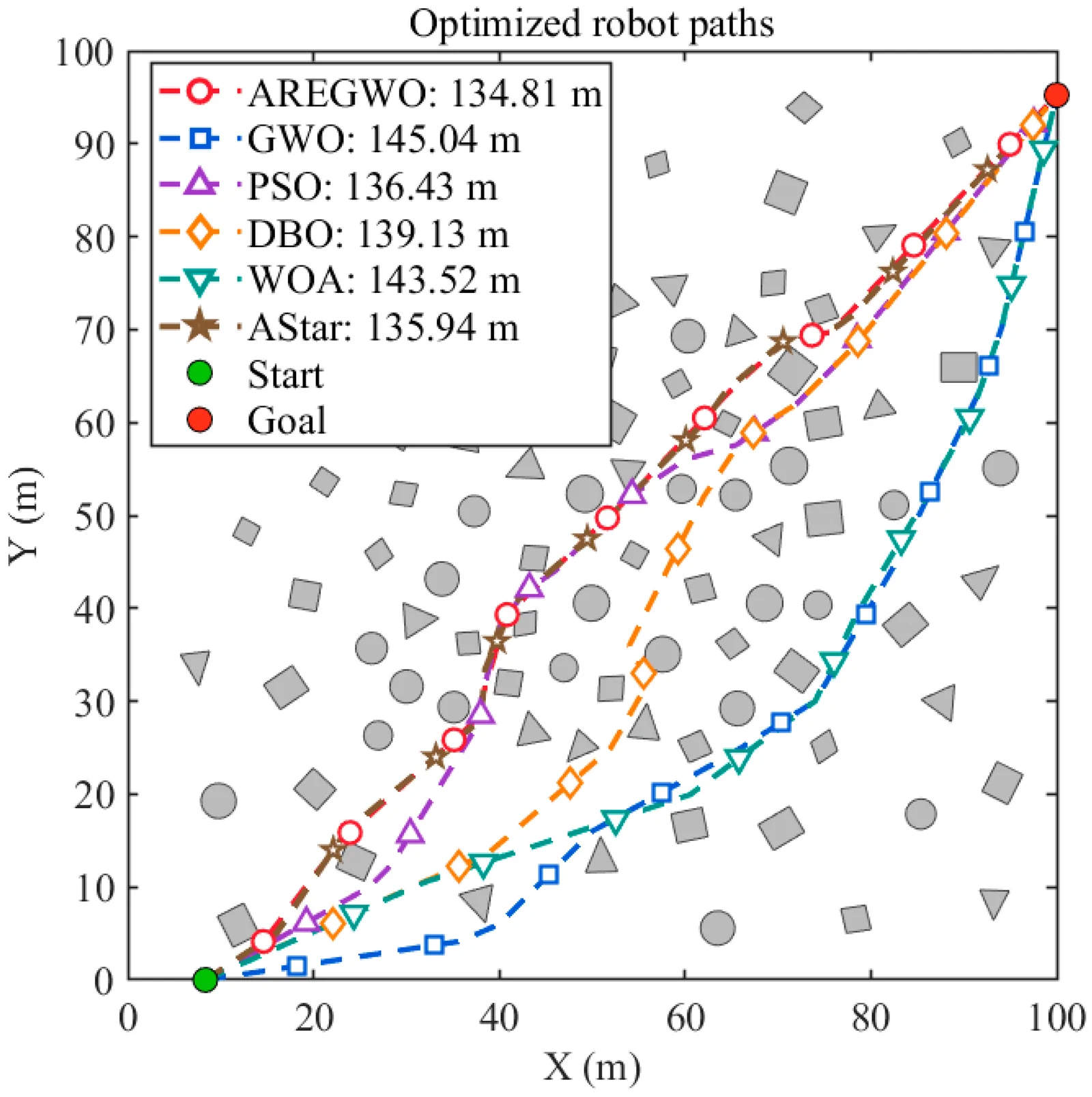
Comparative path-planning results on Random Map 3.

**Figure 6:**
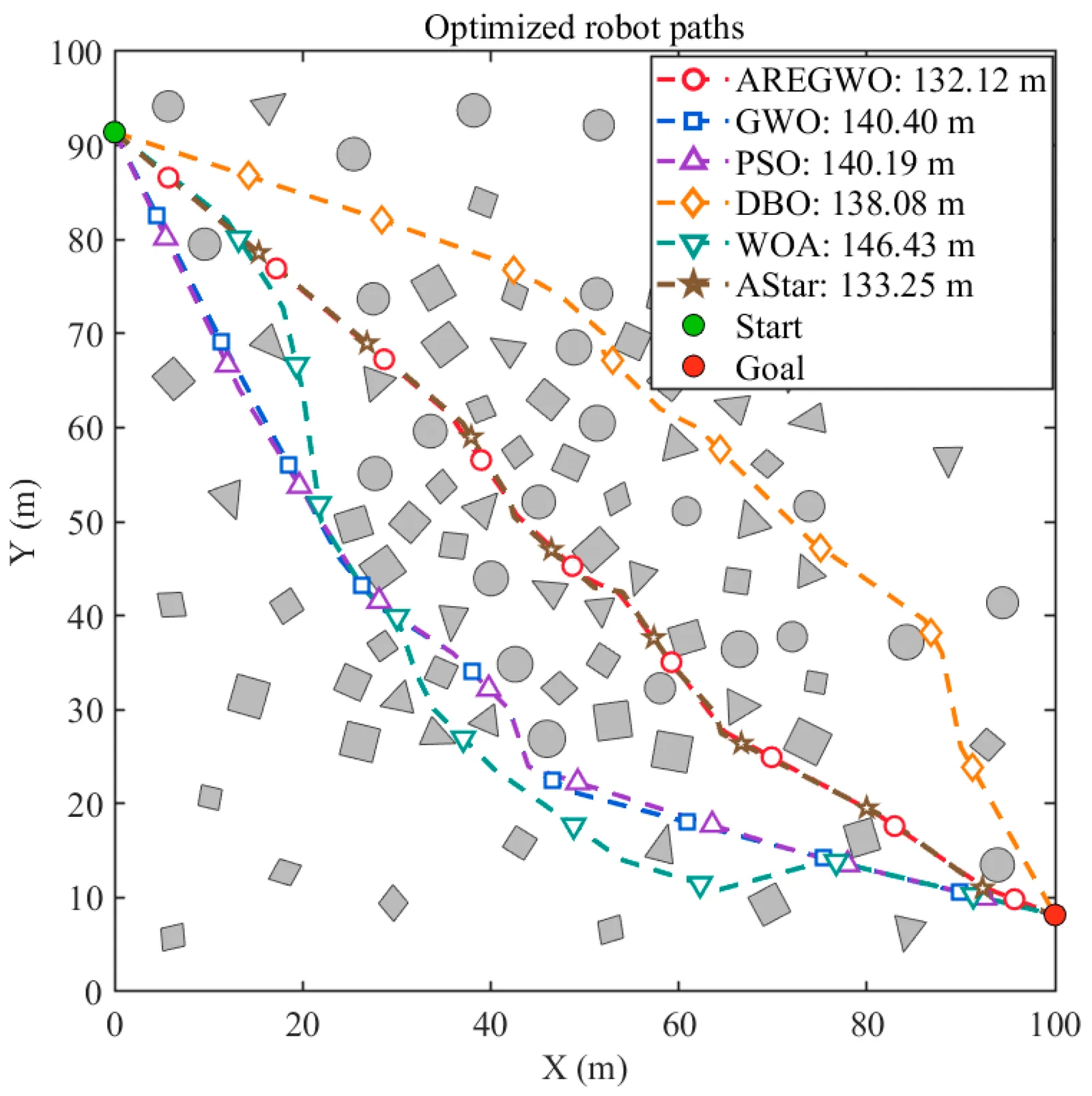
Comparative path-planning results on Random Map 4.

**Figure 7:**
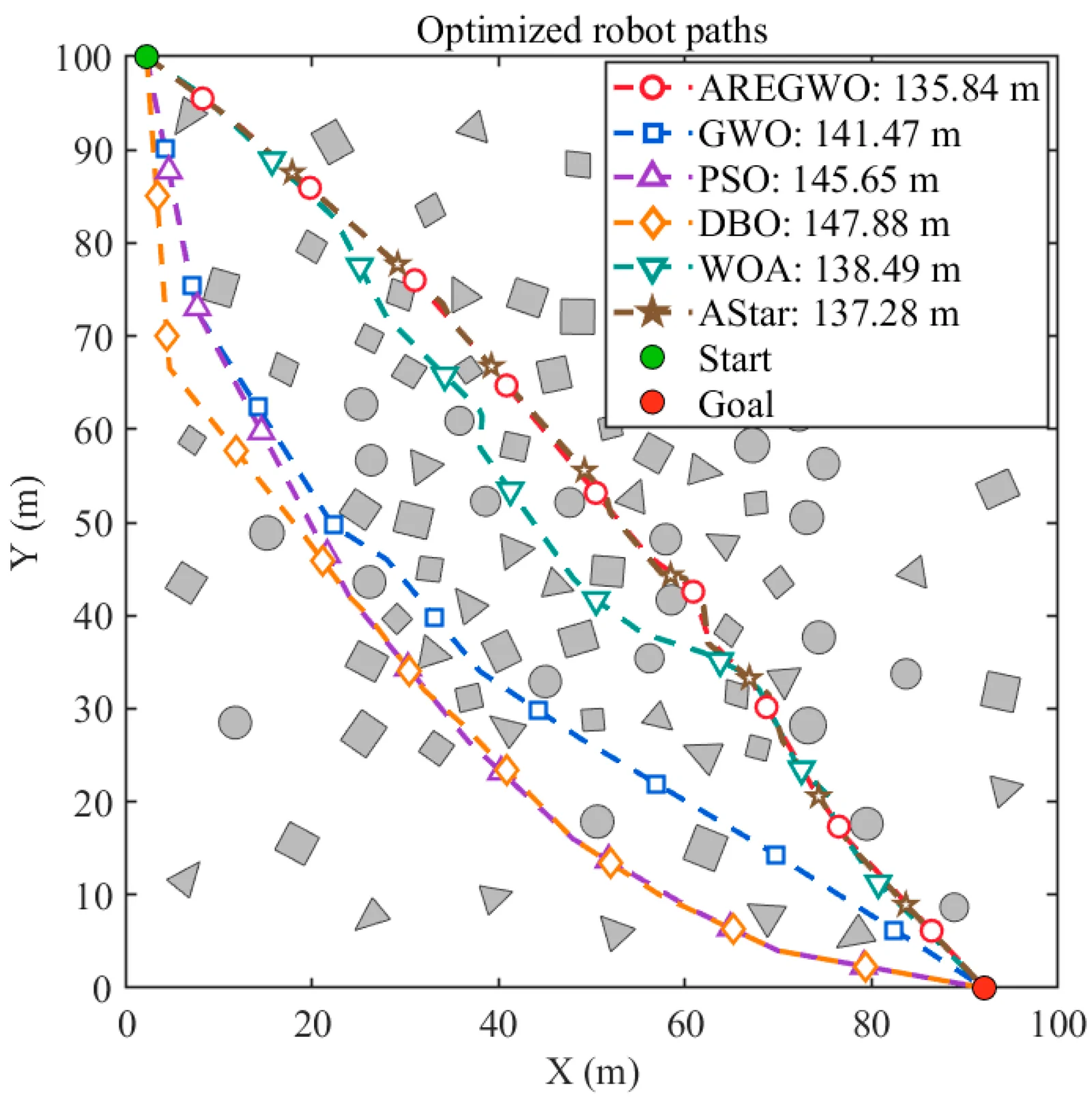
Comparative path-planning results on Random Map 5.

**Figure 8:**
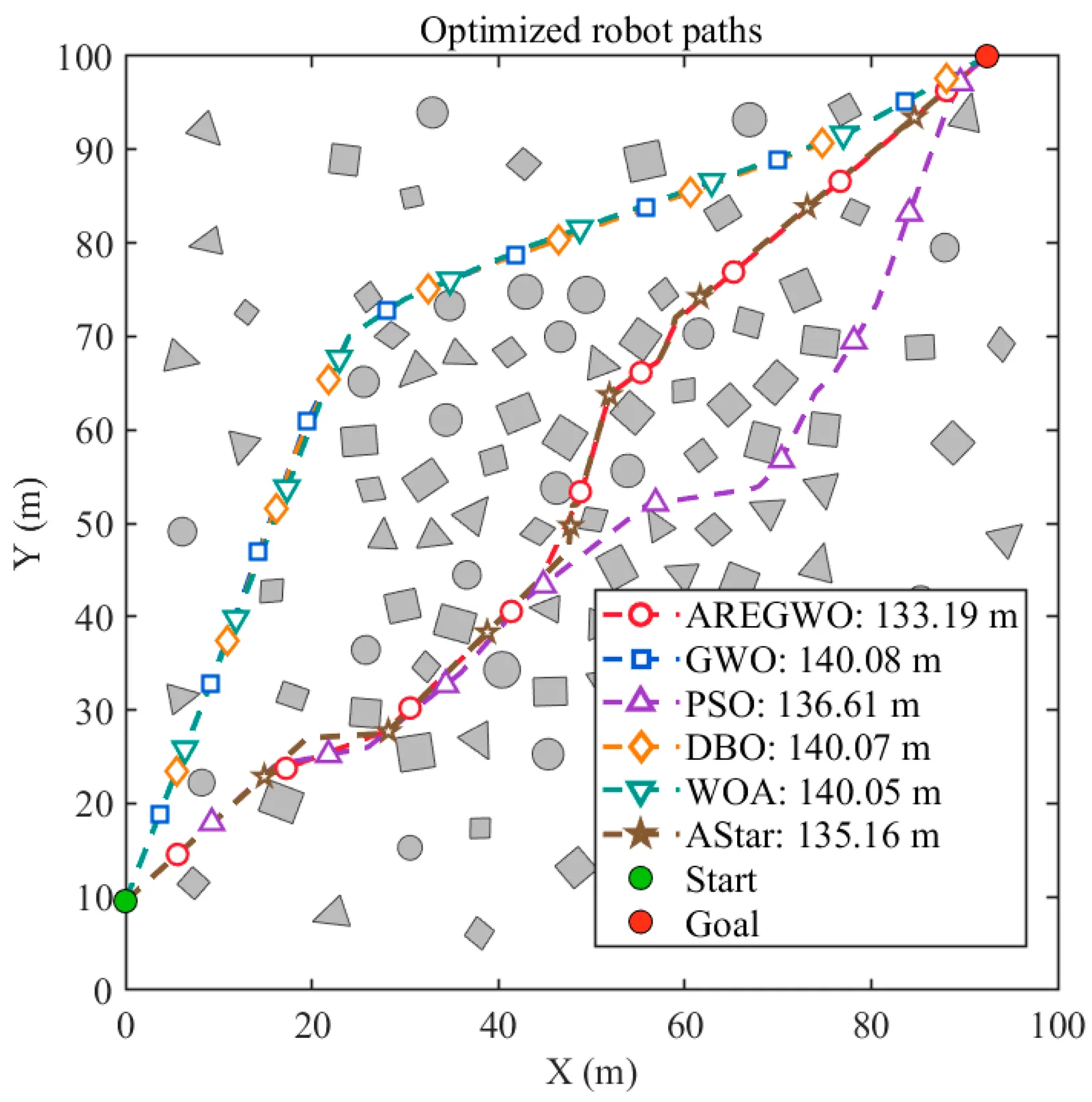
Comparative path-planning results on Random Map 6.

Figure 3, Figure 4, Figure 5, Figure 6, Figure 7 and Figure 8 present one randomly selected run from the 30 independent trials for Random Maps 1–6, respectively, and are intended to illustrate the geometric characteristics of the generated routes rather than represent the statistical performance of the algorithms. As shown in these figures, all six methods are able to construct collision-free routes under different obstacle distributions and start–goal orientations. The resulting trajectories also exhibit clear differences in route geometry: GWO, PSO, DBO, and WOA produce varying degrees of lateral detour depending on the local obstacle configuration, whereas AREGWO generally follows a more compact progression toward the goal. A* provides a stable graph-based feasible route and serves as an additional deterministic reference. The six representative path-planning examples are shown in Figure 3, Figure 4, Figure 5, Figure 6, Figure 7 and Figure 8.

The statistical results over 30 independent runs are summarized in Table 5. In terms of path length, AREGWO obtains the lowest mean value on all six maps, with mean lengths of 132.43, 137.92, 134.81, 132.14, 135.83, and 133.15 m for Maps 1–6, respectively. The corresponding six-map average is 134.38 m, compared with 140.88 m for GWO, 139.41 m for PSO, 141.63 m for DBO, 140.23 m for WOA, and 135.84 m for A*. Thus, averaged across the six environments, the path generated by AREGWO is approximately 4.61%, 3.61%, 5.12%, and 4.17% shorter than those obtained by GWO, PSO, DBO, and WOA, respectively, and approximately 1.08% shorter than the A* result. Moreover, the standard deviation of AREGWO path length remains between 0.01 and 0.05 m across the six maps, indicating highly consistent route-length performance over repeated runs.

The computation-time results reveal a different trade-off. A* is consistently the fastest method, with an average computation time of approximately 6.66 s across the six environments. AREGWO requires an average of approximately 31.64 s, which is higher than A* because additional continuous optimization is performed, but substantially lower than the average times of GWO, PSO, DBO, and WOA, which are approximately 71.15, 70.67, 65.88, and 66.49 s, respectively. Therefore, the shorter paths obtained by AREGWO are achieved without the computational cost observed for the other population-based methods. All six algorithms achieve a success rate of 100% on every map, showing that the prescribed collision and feasibility constraints can be satisfied reliably under the tested environments.

For the minimum safety distance, all reported routes satisfy the prescribed safety-clearance requirement. AREGWO maintains a minimum safety distance of approximately 1.057 m on all six maps, with almost negligible variation among repeated runs. GWO, DBO, WOA, and A* occasionally maintain slightly larger clearances, reaching approximately 1.10 m in some environments. This result should not be interpreted as a disadvantage of AREGWO, because the current optimization formulation minimizes the route length subject to the prescribed safety constraint rather than maximizing obstacle clearance. The AREGWO solutions therefore tend to operate close to the admissible safety boundary while reducing unnecessary detours. In contrast, methods producing larger safety distances may also generate longer routes. This observation illustrates the trade-off between path compactness and additional obstacle clearance in the tested planning problem.

The six-map experiments provide a broader assessment of AREGWO than the original three-environment evaluation. AREGWO achieves the shortest mean path length in all six environments, maintains a 100% success rate, satisfies the prescribed safety-clearance constraint, and requires substantially less computation time than the other population-based optimizers. Although A* remains the fastest method and several comparison algorithms maintain slightly larger obstacle clearances in individual environments, AREGWO provides a favorable overall balance among route compactness, computational efficiency, feasibility, and safety. These results demonstrate the applicability of AREGWO across the tested synthetic 2D path-planning environments with different obstacle distributions and start–goal configurations.

## 5. Football Pass-Quality Prediction

### 5.1. Football Pass-Quality Prediction Model

Football pass-quality prediction is formulated as a supervised regression problem that estimates the quality of an open-play pass from information available when the pass is initiated. The prediction inputs are derived from structured football event records and are organized into three groups: contextual features, pressure-related features, and historical cooperation features. For the *i*-th pass, the input vector is expressed as

(21) $$x_{i}={\left[{\left(x_{i}^{\mathrm{ctx}}\right)}^{T},{\left(x_{i}^{\mathrm{prs}}\right)}^{T},{\left(x_{i}^{\mathrm{his}}\right)}^{T}\right]}^{T}$$

where **x***_i_* denotes the complete input feature vector, $x_{i}^{\mathrm{ctx}}$ contains contextual information, including the pass starting position, match time, and current score difference; $x_{i}^{\mathrm{prs}}$ contains pressure information available at pass initiation; and $x_{i}^{\mathrm{his}}$ contains historical interaction information derived exclusively from preceding events. This grouping preserves the structured nature of football event data while separating the spatial, contextual, and historical information used by the prediction model.

To avoid post-event information entering the prediction process, the observed pass endpoint, realized pass length, forward progression, pass angle, completion outcome, and receipt-related information are not included in **x***_i_*. These variables are available only after the passing action has been realized and they are therefore used, when required, to construct the reference target rather than for model input. Similarly, derived interactions involving realized pass geometry are excluded. This separation ensures that the prediction model uses only information that can be known at the time of the passing decision.

Historical cooperation features describe previous passing interactions between the passer and the intended recipient. The events are arranged chronologically, and only interactions occurring before the current pass are included. For a given passer–recipient pair, the historical success rate is calculated as follows:

(22) $$r_{i}=\frac{n_{i}^{\mathrm{suc}}}{n_{i}^{\mathrm{tot}}+\varepsilon }$$

where *r_i_* is the historical success rate associated with the *i*-th passing action, $n_{i}^{\mathrm{suc}}$ is the number of preceding successful passes between the corresponding passer and intended recipient, $n_{i}^{\mathrm{tot}}$ is their total number of preceding passes, and *ε* is a small positive constant introduced to avoid division by zero. Pair-specific historical features are used only when the intended recipient is available at the prediction time; recipient information inferred from the realized passing outcome is not used.

A binary completion indicator alone cannot adequately describe pass quality because passes with the same completion outcome may differ substantially in spatial progression, execution difficulty, and tactical contribution. Therefore, a continuous reference score is constructed from several complementary components. Before score construction, the continuous quantities are normalized using the training data. For a generic variable *z_i_*, min–max normalization is defined as

(23) $${\tilde{z}}_{i}=\frac{z_{i}-z_{\min}}{z_{\max}-z_{\min}+\varepsilon }$$

where ${\tilde{z}}_{i}$ is the normalized value, and *z*_min_ and *z*_max_ are the minimum and maximum values of *z_i_* in the training set, respectively. The same normalization parameters are subsequently applied to the validation and testing data.

The unnormalized pass-quality reference score is then defined as

(24) $$q_{i}=c_{1}s_{i}+c_{2}{\bar{g}}_{i}+c_{3}{\bar{l}}_{i}+c_{4}{\bar{r}}_{i}-c_{5}d_{i}$$

where *q_i_* denotes the reference score of the *i*-th pass; *s_i_* is the pass-completion indicator; ${\bar{g}}_{i}$ is the normalized forward progression toward the opponent’s goal; ${\bar{l}}_{i}$ is the normalized realized pass length; ${\bar{r}}_{i}$ is the normalized historical passer–recipient success rate; and *d_i_* is the pressure indicator, which equals 1 when the passer is under defensive pressure and 0 otherwise. The coefficients were fixed as *c*_1_ = 0.35, *c*_2_ = 0.20, *c*_3_ = 0.15, *c*_4_ = 0.15, *c*_5_ = 0.15 throughout all experiments. These weights were specified before model training to provide a fixed reference target and were not optimized using the test data. The weighting scheme gives primary consideration to pass completion while incorporating progression, passing distance, historical cooperation, and defensive pressure as complementary contextual factors. Its purpose is to construct a continuous reference target for model comparison rather than to define a universally valid tactical-value metric.

The final regression target is obtained by normalizing *q_i_*:

(25) $$y_{i}=\frac{q_{i}-q_{\min}}{q_{\max}-q_{\min}+\varepsilon }$$

where *y_i_* is the normalized pass-quality target, and *q*_min_ and *q*_max_ are determined from the training data. It should be emphasized that *y_i_* represents a predefined composite reference score rather than an independently measured tactical ground truth. Accordingly, the regression performance reported in this study should be interpreted as the model’s ability to reproduce the predefined composite reference score on unseen data, rather than as agreement with an independently observed tactical ground truth. A high R^2^ or a low prediction error therefore indicates strong predictive consistency with the adopted scoring formulation, but it does not by itself establish the external validity of the score as an absolute measure of pass quality. The observed pass outcome and realized geometry contribute to target construction but are excluded from the prediction inputs. Pressure and historical cooperation information can appear in both the input representation and the scoring rule when they are already available at pass initiation; such overlap does not introduce future information into the model.

Based on the constructed input–target pairs, football pass-quality prediction is expressed as the nonlinear mapping

(26) $${\hat{y}}_{i}=f(x_{i};\theta )$$

where ${\hat{y}}_{i}$ is the predicted pass-quality score, *f*(·) denotes the neural tabular regression model, and *θ* represents its trainable parameters. The model therefore learns the relationship between the information available at pass initiation and the subsequently constructed continuous reference score. The model parameters are learned from the training data, whereas the external hyperparameters controlling the model architecture and training process are optimized by AREGWO.

### 5.2. AREGWO-Based TabM Optimization

The football pass-quality prediction task defined in Section 5.1 is further coupled with AREGWO to form an automatic hyperparameter optimization framework. TabM is adopted as the regression model because the football predictors are primarily structured tabular variables rather than image or sequence inputs. In the proposed framework, AREGWO does not directly update the internal trainable weights of TabM. Instead, each search agent represents one candidate hyperparameter configuration, while the corresponding TabM model is trained on the training subset and evaluated on the validation subset. The resulting validation error is then returned to AREGWO as the fitness value. This preserves a clear separation between neural-network training and outer-level swarm optimization.

Let the normalized hyperparameter candidate searched by AREGWO be written as

(27) $$h=\left[h_{1},h_{2},\ldots ,h_{D}\right],0\leq h_{j}\leq 1$$

where **h** denotes a candidate hyperparameter vector, *D* is the number of hyperparameters to be optimized, and *h_j_* is the normalized value of the *j*-th hyperparameter. The optimized configuration includes the principal TabM training and structural parameters, such as learning rate, weight decay, embedding dimension, hidden dimension, dropout rate, and ensemble-related parameters.

Because different TabM hyperparameters have different numerical forms, the normalized AREGWO variables are decoded before model training. For a continuous parameter, the decoded value is obtained by linear interval mapping; logarithmic mapping is used for parameters spanning several orders of magnitude, such as learning rate and weight decay; and integer-valued parameters are rounded to feasible discrete values. These three cases can be summarized as

(28) $$p_{j}=\left\{\begin{array}{l} p_{j}^{\min}+h_{j}(p_{j}^{\max}-p_{j}^{\min}),\mathrm{continuous}\\ 10^{\log_{10}p_{j}^{\min}+h_{j}(\log_{10}p_{j}^{\max}-\log_{10}p_{j}^{\min})},\log\;\mathrm{arithmic}\\ \mathrm{round}\left[p_{j}^{\min}+h_{j}(p_{j}^{\max}-p_{j}^{\min})\right],\mathrm{int}\;\mathrm{eger} \end{array}\right.$$

where *p_j_* is the decoded value of the j-th TabM hyperparameter, and $p_{j}^{\min}$ and $p_{j}^{\max}$ are its lower and upper search bounds, respectively. This mixed decoding strategy enables AREGWO to search continuous, logarithmic, and discrete parameters within the same optimization process.

For a decoded hyperparameter configuration **p**, TabM is trained using the training subset defined for the football prediction task. The internal model parameters *θ* introduced in Equation (26) are obtained by minimizing the regression error on the training samples:

(29) $$\theta *(p)=\arg\min_{\theta }\frac{1}{N_{\mathrm{tr}}}{\sum_{i\in o_{\mathrm{tr}}}\left[y_{i}-f(x_{i};\theta ,p)\right]}^{2}$$

where *O_tr_* denotes the training index set, *N_tr_* is the number of training samples, and *θ* * (**p**) represents the trained TabM parameters obtained under hyperparameter configuration **p**. Therefore, for every AREGWO candidate, the internal TabM model is trained independently before its predictive performance is evaluated.

The validation root mean squared error is used as the fitness function of AREGWO:

(30) $$J_{\mathrm{val}}(h)=\sqrt{\frac{1}{N_{\mathrm{val}}}{\sum_{i\in o_{\mathrm{val}}}\left[y_{i}-f(x_{i};\theta *(p(h)),p(h))\right]}^{2}}$$

where *J_val_*(**h**) denotes the fitness value associated with candidate **h**, *O_val_* is the validation index set, and *N_val_* is the number of validation samples. A smaller value of *J_val_* indicates a more effective TabM hyperparameter configuration.

Accordingly, the outer-level hyperparameter optimization is expressed as

(31) $$h*=\arg\min_{h\in {\left[0,1\right]}^{D}}J_{\mathrm{val}}(h),p=p(h*)$$

where h* is the optimal normalized candidate found by AREGWO and p* is the corresponding TabM hyperparameter configuration. The overall procedure therefore forms a bilevel learning framework: the inner level trains the TabM parameters for a given hyperparameter configuration, whereas the outer level uses AREGWO to minimize the validation error over the hyperparameter search space.

After the optimal configuration is obtained, the final TabM model is retrained using the training and validation data together and then applied to the independent testing subset. No testing samples are used during the AREGWO search, so the test results remain independent of hyperparameter selection.

### 5.3. Experimental Validation

The football data used in this study were derived from the publicly available StatsBomb Open Data repository. Specifically, event data from the Spanish La Liga (competition ID 11) were collected from six consecutive seasons: 2015/2016, 2016/2017, 2017/2018, 2018/2019, 2019/2020, and 2020/2021, corresponding to season IDs 27, 2, 1, 4, 42, and 90, respectively. After match extraction and data cleaning, 231 matches were included. The event fields were processed according to the StatsBomb Data Specification v1.1. The original data are distributed as JSON files through the StatsBomb Open Data repository and were accessed under its public-use terms, which require attribution to StatsBomb [36].

For sample construction, only Pass events occurring under Regular Play were retained. Goalkeeper passes and restart situations, including corners, free kicks, throw-ins, and kick-offs, were excluded to reduce tactical heterogeneity. Events with invalid or missing variables required for feature construction were removed, after which contextual, pressure-related, and historical passer–recipient features were derived from the ordered event streams. The processed modeling dataset was capped at 100,000 eligible open-play passes for computational consistency; specifically, the first 100,000 eligible samples in the processed event order were retained, rather than applying outcome-based or class-balanced sampling. The samples were subsequently ordered temporally and divided into training, validation, and testing subsets using a 70%/15%/15% split. The same partitions were used for all compared models within each experiment, and the testing subset was not involved in model training or AREGWO-based hyperparameter optimization.

#### 5.3.1. Prediction Performance

**Figure 9:**
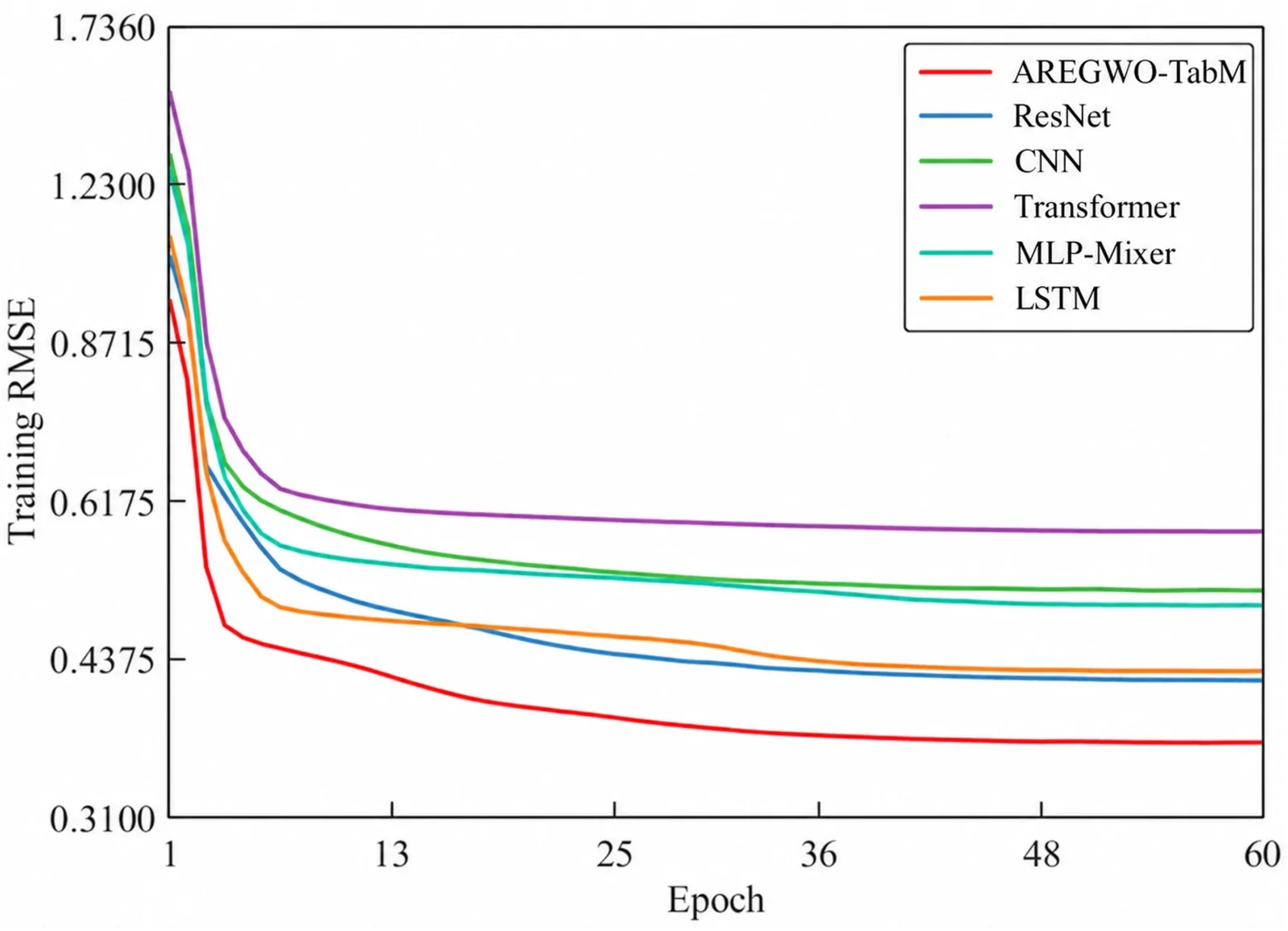
Training-curve comparison of AREGWO-TabM and baseline deep learning models.

**Figure 10:**
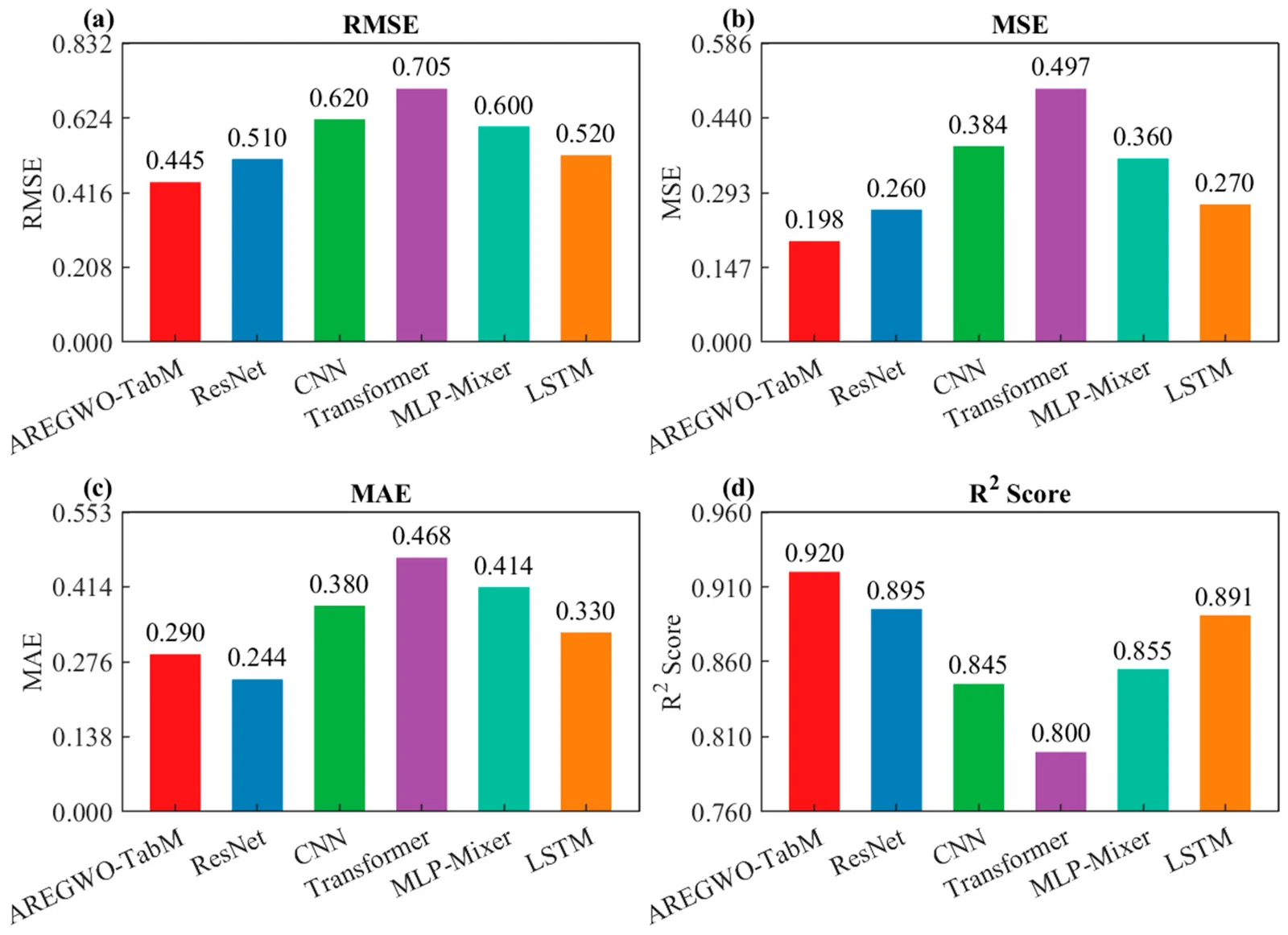
Comparison of regression evaluation metrics among different prediction models. (**a**) RMSE; (**b**) MSE; (**c**) MAE; (**d**) R^2^ Score.

**Figure 11:**
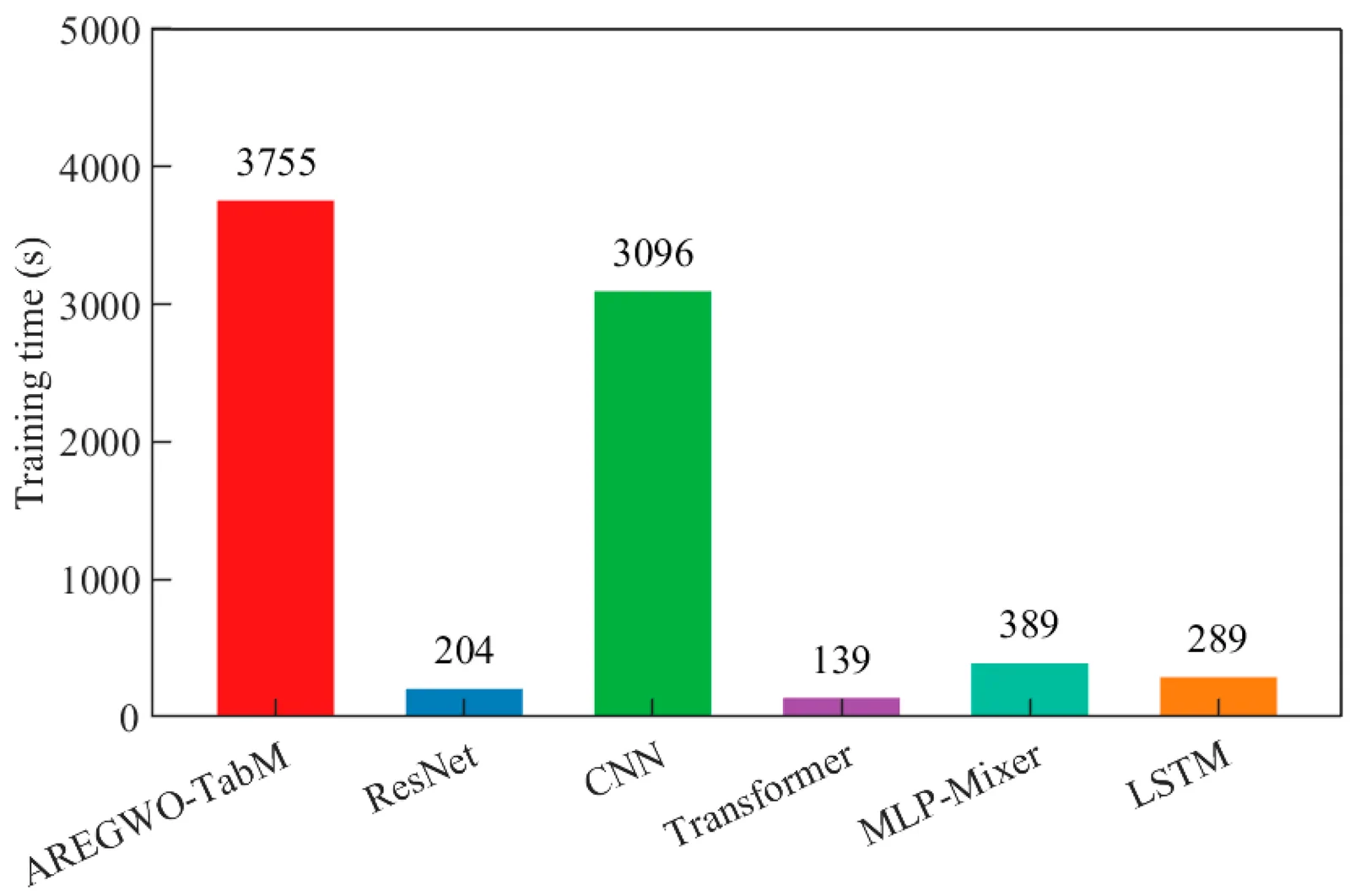
Training time comparison of AREGWO-TabM and baseline models.

**Figure 12:**
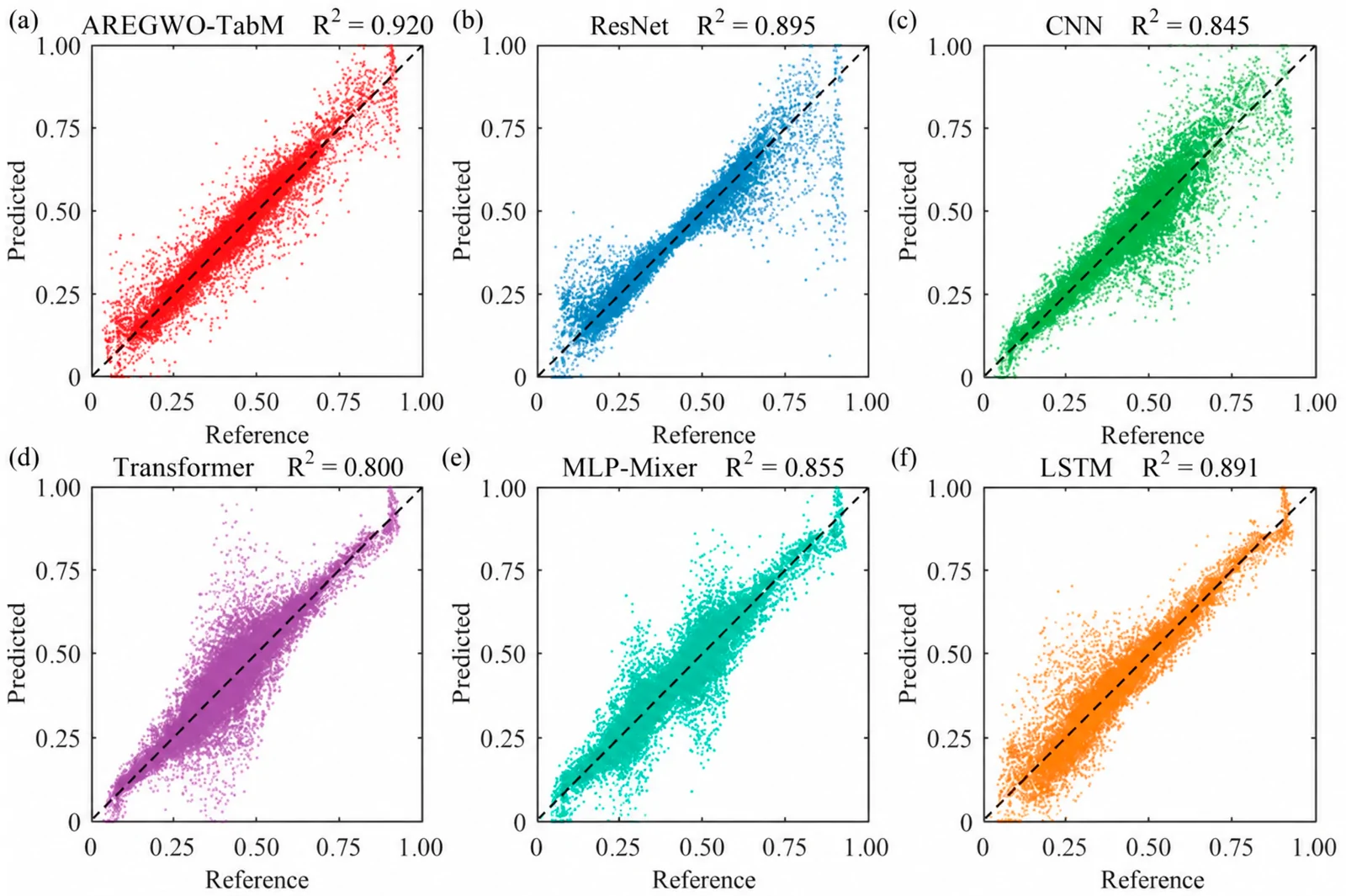
Predicted versus reference pass-quality scores obtained by different models. (**a**) AREGWO-TabM; (**b**) ResNet; (**c**) CNN; (**d**) Transformer; (**e**) MLP-Mixer; (**f**) LSTM.

To evaluate the effectiveness of AREGWO-TabM for football pass-quality regression, five representative neural-network models were selected as comparison methods: ResNet, CNN, Transformer, MLP-Mixer, and LSTM. All models used the same input features and data partitions, and their performance was assessed from four perspectives: training convergence, regression accuracy, computational cost, and agreement between predicted and reference pass-quality scores. Accordingly, Figure 9 presents the training RMSE curves, Figure 10 compares RMSE, MSE, MAE, and R^2^, Figure 11 reports the training time, and Figure 12 compares the predicted and reference pass-quality scores.

As shown in Figure 9, all models exhibit a rapid reduction in training RMSE during the initial learning stage, indicating that the structured football features contain sufficient information for learning the pass-quality regression relationship. However, clear differences emerge as training proceeds. AREGWO-TabM maintains a continuous reduction in RMSE and reaches a lower final error level than the five baseline models. By comparison, CNN, Transformer, and MLP-Mixer enter relatively flat regions earlier, whereas ResNet and LSTM achieve lower errors than these three models but remain above AREGWO-TabM at the end of training. The convergence behavior indicates that the AREGWO-derived hyperparameter configuration enables TabM to reach a lower-error region during model fitting.

Figure 10 compares the regression performance of the six models using RMSE, MSE, MAE, and R^2^. AREGWO-TabM achieves the lowest RMSE and MSE, with values of 0.445 and 0.198, respectively. The corresponding RMSE values for ResNet, CNN, Transformer, MLP-Mixer, and LSTM are 0.510, 0.620, 0.705, 0.600, and 0.520, while their MSE values are 0.260, 0.384, 0.497, 0.360, and 0.270, respectively. For MAE, ResNet achieves the lowest value of 0.244, followed by AREGWO-TabM (0.290), LSTM (0.330), CNN (0.380), MLP-Mixer (0.414), and Transformer (0.468). In terms of goodness of fit, AREGWO-TabM obtains the highest R^2^ of 0.920, compared with 0.895, 0.845, 0.800, 0.855, and 0.891 for ResNet, CNN, Transformer, MLP-Mixer, and LSTM, respectively.

The differences among these metrics also provide useful information about the error distribution. MAE weights individual prediction errors linearly, whereas RMSE assigns greater influence to larger deviations. The lower RMSE of AREGWO-TabM, despite its higher MAE than ResNet, therefore indicates that the proposed model is particularly effective in limiting relatively large prediction deviations. Together with the higher R^2^, this suggests a favorable overall fit to the continuous pass-quality reference score rather than uniform superiority under every error measure.

Figure 11 presents the computational cost of the different prediction models. AREGWO-TabM requires the longest training time, approximately 3755 s, because its computational procedure contains an additional outer-loop hyperparameter search in which multiple TabM configurations must be trained and validated before the final model is obtained. CNN also incurs a relatively high training cost of 3096 s, whereas the training times of ResNet, Transformer, MLP-Mixer, and LSTM are 204, 139, 389, and 289 s, respectively. Therefore, the improvement in predictive performance achieved by AREGWO-TabM is accompanied by a substantial increase in offline computational cost. This trade-off is expected for the proposed framework because AREGWO performs repeated model evaluations during hyperparameter optimization, whereas the baseline networks are trained directly under predetermined configurations.

The additional optimization time should therefore be interpreted as an offline model-selection cost rather than as an increase in the computational complexity of each individual prediction. Once the optimal TabM configuration has been identified and the final model has been trained, AREGWO is no longer involved in generating pass-quality predictions. Consequently, the principal computational overhead occurs during model development and optimization rather than during subsequent inference.

Figure 12 further compares the predicted scores with the reference pass-quality values. The diagonal line represents perfect agreement between prediction and reference. All models reproduce the overall variation in the target variable, but their prediction dispersion differs noticeably. The AREGWO-TabM results are more concentrated around the ideal diagonal, consistent with its R^2^ of 0.920. ResNet and LSTM also show relatively close agreement, with R^2^ values of 0.895 and 0.891, respectively. Greater dispersion is observed for MLP-Mixer and CNN, while Transformer shows the weakest correspondence, consistent with its R^2^ of 0.800. These observations agree with the quantitative results in Figure 10 and indicate that AREGWO-TabM captures a larger proportion of the variation in the constructed pass-quality reference score.

The prediction experiments show that AREGWO-TabM provides a favorable balance between regression accuracy and goodness of fit. The proposed model achieves the lowest RMSE and MSE and the highest R^2^ among the evaluated methods, although ResNet obtains a lower MAE. The improvement is accompanied by increased offline computational cost due to AREGWO-based hyperparameter optimization. Taken together, these results indicate that adaptive optimization of TabM hyperparameters improves the prediction of the predefined pass-quality reference score while introducing a clear accuracy–computation trade-off.

#### 5.3.2. AREGWO-TabM Ablation Analysis

To further examine the contribution of AREGWO-based hyperparameter optimization to football pass-quality prediction, an ablation study was conducted using three TabM-based configurations: AREGWO-TabM, GWO-TabM, and TabM without metaheuristic optimization. GWO-TabM replaces the proposed AREGWO with the standard GWO while retaining the same underlying TabM model, thereby providing a direct comparison between the proposed optimizer and its canonical counterpart. The fixed-parameter TabM model does not employ population-based hyperparameter optimization and is included to assess the overall contribution of automatic model configuration. Within this independently conducted ablation experiment, all three configurations use the same prediction task, input features, data partition, and regression target.

**Figure 13:**
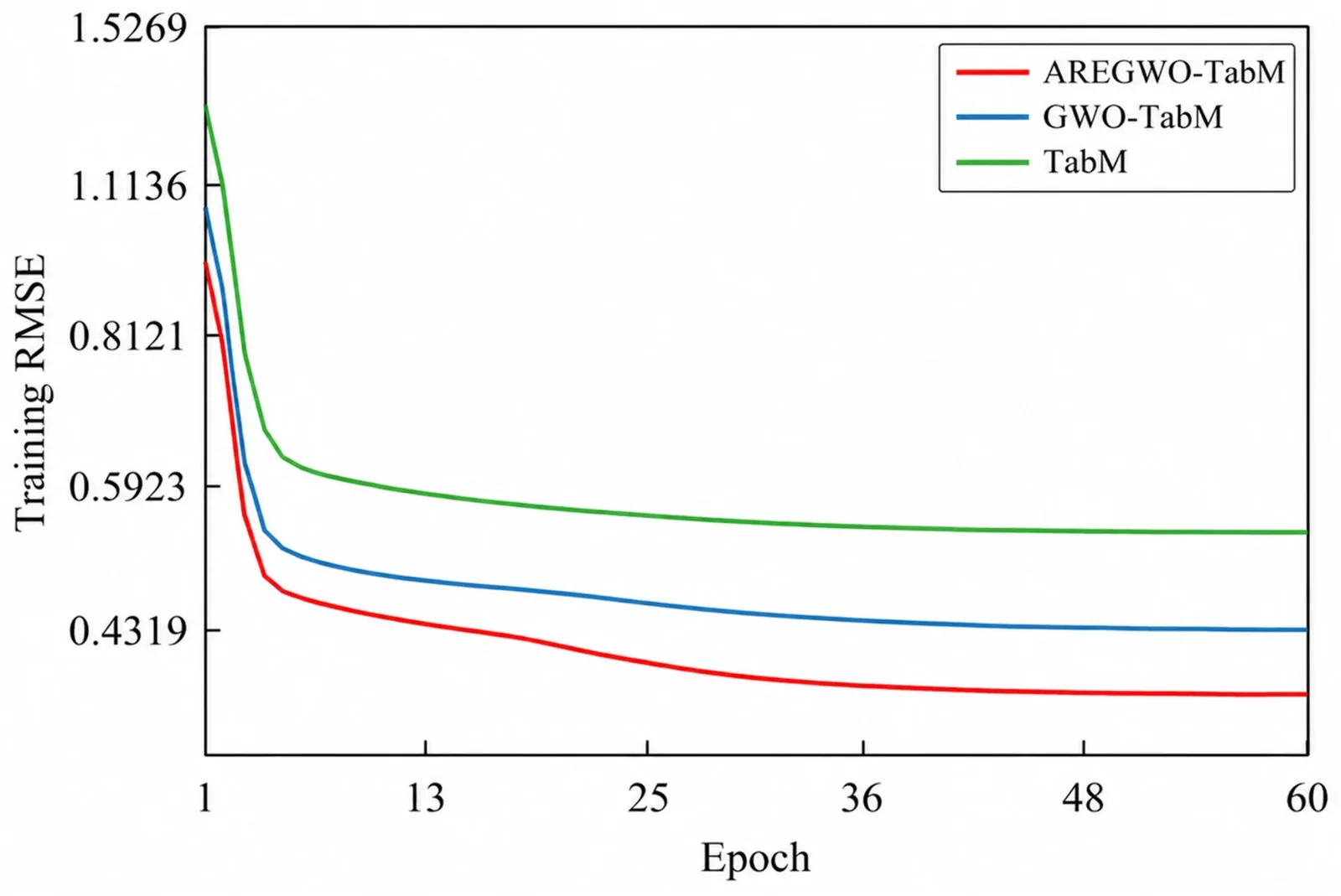
Training-curve comparison in the ablation study.

**Figure 14:**
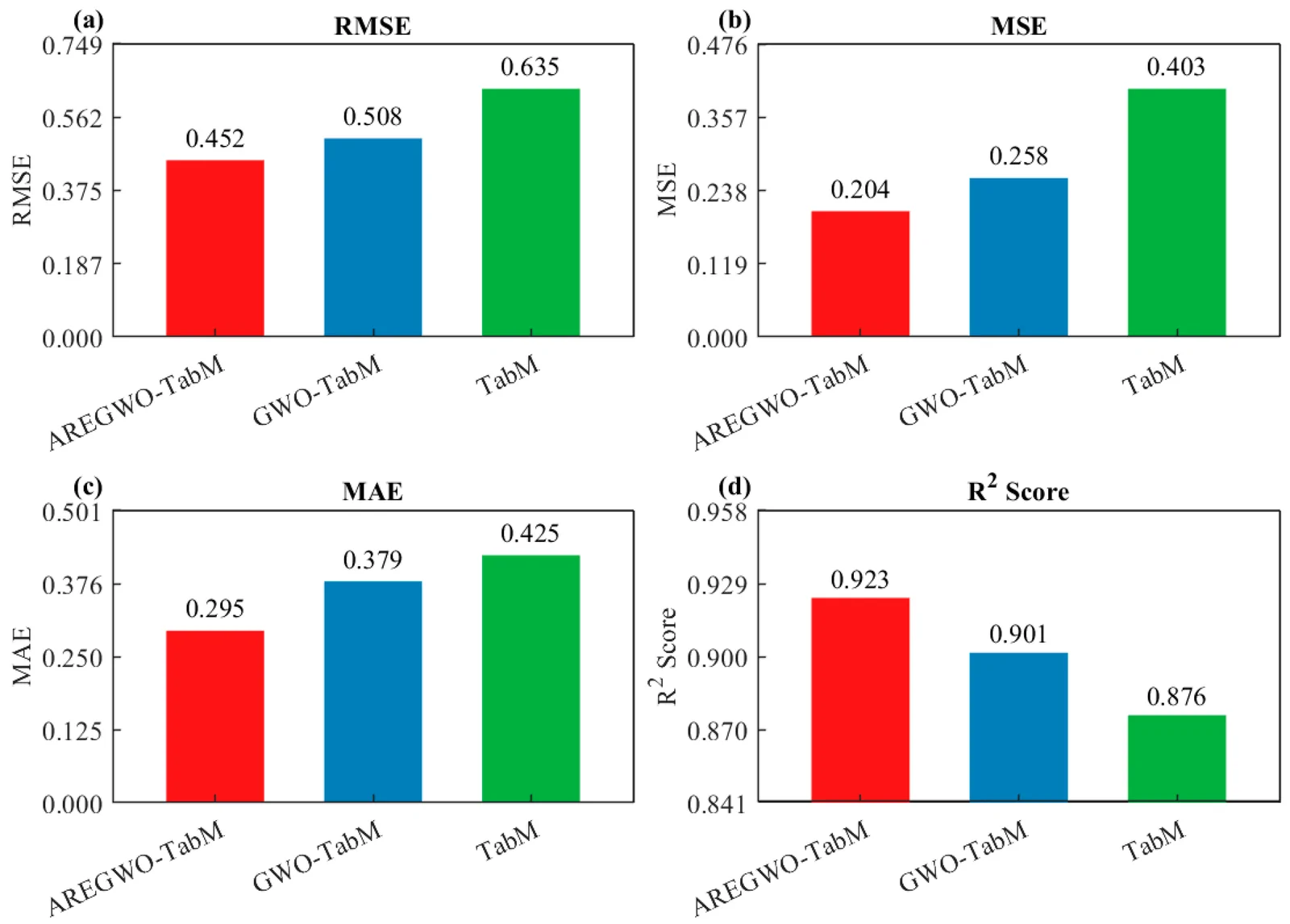
Comparison of regression evaluation metrics in the ablation study. (**a**) RMSE; (**b**) MSE; (**c**) MAE; (**d**) R2 Score.

**Figure 15:**
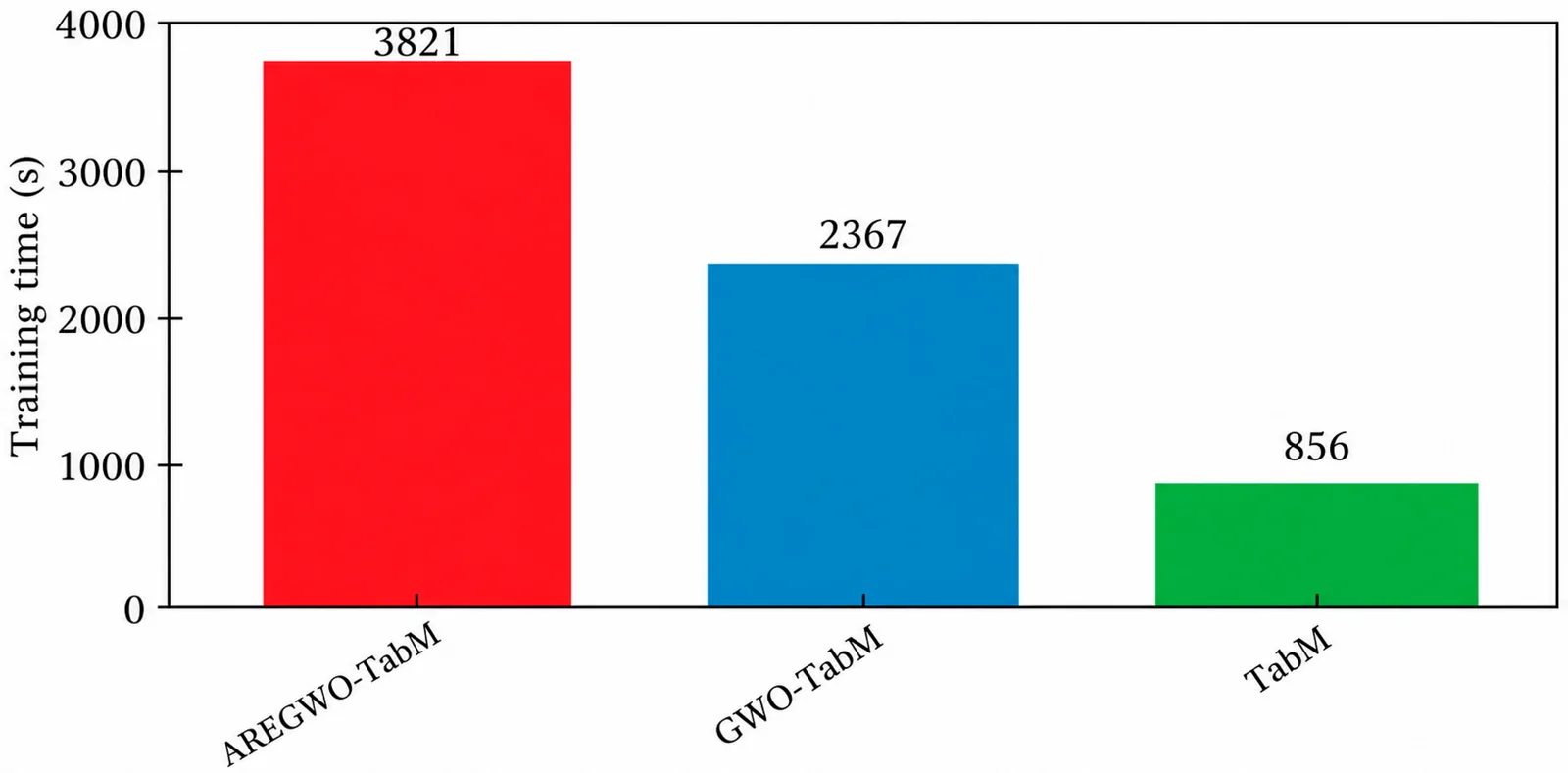
Training-time comparison in the ablation study.

**Figure 16:**
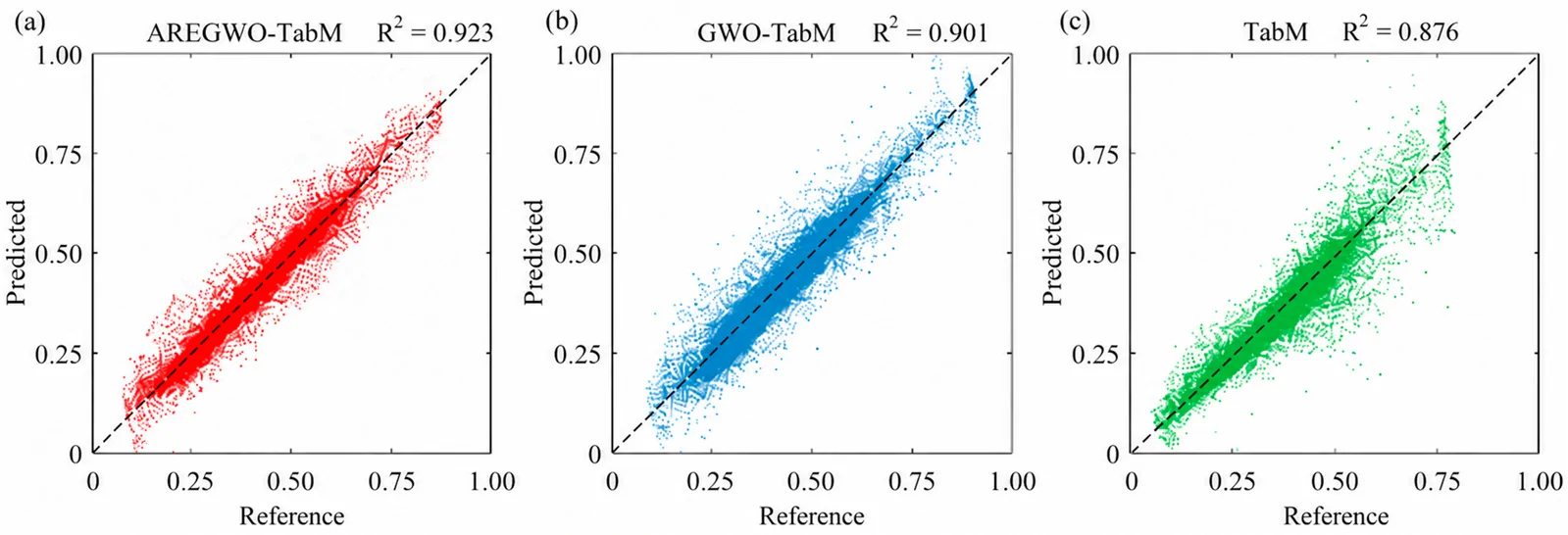
Predicted versus reference pass-quality scores in the ablation study. (**a**) AREGWO-TabM; (**b**) GWO-TabM; (**c**) TabM.

The ablation analysis considers the training convergence, regression accuracy, computational cost, and prediction agreement. Figure 13 compares the training RMSE curves of the three configurations, Figure 14 reports RMSE, MSE, MAE, and R^2^, Figure 15 compares their computational time, and Figure 16 presents the predicted versus reference pass-quality scores.

As shown in Figure 13, all three TabM-based models exhibit a rapid decrease in RMSE during the initial training stage. However, their convergence trajectories gradually diverge as training proceeds. The fixed-parameter TabM and GWO-TabM models reach relatively stable error levels earlier, whereas AREGWO-TabM continues to reduce the training error and maintains a lower RMSE over most of the later training process. This difference indicates that the hyperparameter configuration identified by AREGWO provides a more favorable training condition for TabM than either the fixed configuration or the configuration obtained using standard GWO.

Figure 14 provides a quantitative comparison of the three configurations. AREGWO-TabM achieves an RMSE of 0.452, compared with 0.508 for GWO-TabM and 0.635 for fixed-parameter TabM. These values correspond to RMSE reductions of approximately 11.0% and 28.8%, respectively. A similar pattern is observed for MSE, where AREGWO-TabM achieves 0.204, lower than 0.258 for GWO-TabM and 0.403 for TabM. The corresponding MAE values are 0.295, 0.379, and 0.425, respectively. Thus, unlike the comparison with the heterogeneous baseline networks in Section 5.3.1, AREGWO-TabM obtains the lowest values for all three error metrics in this TabM-based ablation comparison.

The same tendency is observed for goodness of fit. AREGWO-TabM achieves the highest R^2^ of 0.923, compared with 0.901 for GWO-TabM and 0.876 for the fixed-parameter TabM model. The improvement from fixed TabM to GWO-TabM indicates that an automatic hyperparameter search is beneficial to the regression task, while the further improvement from GWO-TabM to AREGWO-TabM shows that the proposed search strategy identifies a more effective TabM configuration than standard GWO under the present experimental setting.

The improvement in predictive performance is accompanied by an increase in offline computational cost. As shown in Figure 15, fixed-parameter TabM requires 856 s, whereas GWO-TabM and AREGWO-TabM require 2367 and 3821 s, respectively. Relative to fixed-parameter TabM, AREGWO-TabM therefore introduces an additional 2965 s of computation, corresponding to approximately 4.46 times the total computational time. This additional cost is associated with an RMSE reduction from 0.635 to 0.452 (28.8%), an MSE reduction from 0.403 to 0.204 (49.4%), an MAE reduction from 0.425 to 0.295 (30.6%), and an increase in R^2^ from 0.876 to 0.923. Thus, the performance gain is obtained at a substantial but quantifiable offline computational cost.

The additional expense mainly arises from repeatedly training and validating candidate TabM configurations during the outer hyperparameter search. Importantly, this cost is incurred during model development rather than during subsequent prediction. Once the optimal hyperparameter configuration has been identified and the final TabM model has been trained, AREGWO is no longer involved in inference. Therefore, the proposed framework is more suitable for offline model development and periodic model updating than for applications requiring frequent online hyperparameter re-optimization.

Figure 16 further illustrates the agreement between the predicted and reference pass-quality scores. The diagonal line represents perfect agreement between the two quantities. Predictions obtained by AREGWO-TabM are more closely distributed around this reference line, while progressively larger deviations are observed for GWO-TabM and fixed-parameter TabM. This graphical pattern is consistent with their corresponding R^2^ values of 0.923, 0.901, and 0.876, respectively, and with the RMSE results shown in Figure 14. The comparison therefore provides additional evidence that the performance improvement of AREGWO-TabM is associated not only with the TabM architecture itself but also with the optimized hyperparameter configuration identified by AREGWO.

The ablation results show two successive improvements. Introducing standard GWO improves the predictive performance of TabM relative to a fixed hyperparameter configuration, demonstrating the value of automatic hyperparameter optimization. Replacing standard GWO with AREGWO further reduces RMSE, MSE, and MAE while increasing R^2^, indicating that the proposed adaptive search strategy provides additional optimization benefit. These gains are achieved at the expense of greater offline computational time, highlighting the trade-off between search cost and predictive accuracy in the proposed framework.

### 5.4. Implications for Football Analytics and Education

**Figure 17:**
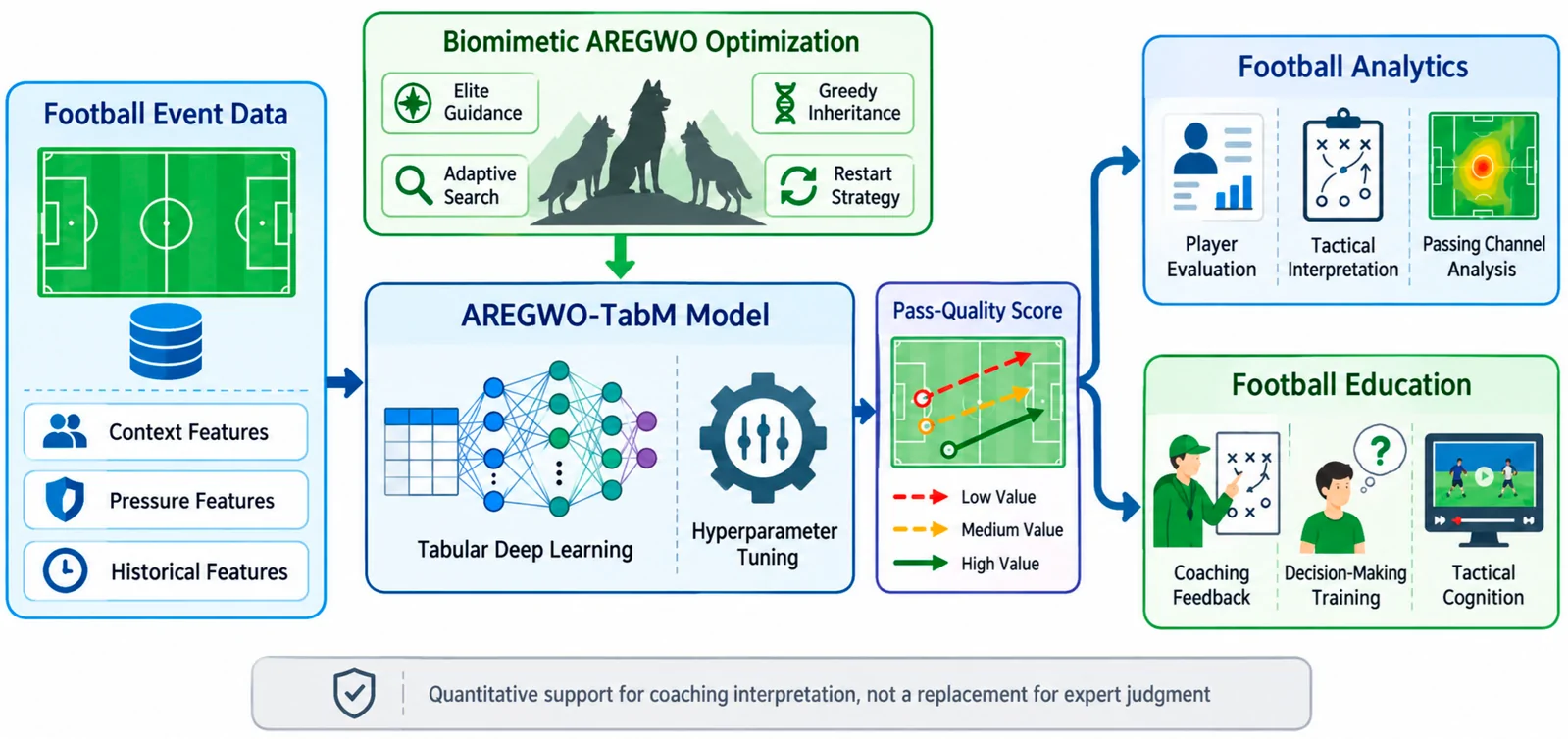
Implications of AREGWO-TabM for football analytics and education.

The proposed AREGWO-TabM framework provides a quantitative approach to football pass analysis by extending evaluation beyond binary pass completion toward continuous pass-quality estimation. As illustrated in Figure 17, contextual, pressure-related, and historical cooperation information available at pass initiation is integrated through the TabM regression model, while AREGWO is used to identify an effective hyperparameter configuration. The resulting pass-quality score provides a more differentiated representation of passing actions than isolated indicators such as completion status or passing distance. Passes with the same completion outcome can receive different reference scores according to their forward progression, execution distance, pressure condition, and historical passer–recipient interaction. This enables the model to distinguish between passing actions that would otherwise appear identical under a binary success indicator.

From an application perspective, the proposed framework also has potential value for football training and education. Tactical concepts such as forward progression, passing under pressure, spatial awareness, and passer–recipient cooperation are often interpreted qualitatively during coaching and instruction. The continuous pass-quality score provides a quantitative representation that can complement such qualitative assessment. For example, two successfully completed passes may receive different scores because of differences in progression, passing distance, pressure condition, and historical cooperation. This type of quantitative feedback may assist coaches and learners in comparing passing actions under different contextual conditions.

Nevertheless, the predicted pass-quality score should be interpreted as a decision-support indicator rather than an absolute measure of player ability or tactical value. The reference score used in this study is constructed from predefined components, while actual football decisions may additionally depend on tactical instructions, teammate and opponent movements, available space, and other information not represented in the current event data. Accordingly, the high predictive performance reported in Section 5.3 reflects consistency with the constructed reference target rather than validation against an independent tactical ground truth. Therefore, model outputs are more appropriately used together with coaching expertise and video-based analysis. Although AREGWO-TabM incurs a higher offline optimization cost, this overhead can potentially be reduced through parallel or distributed computing and does not affect routine inference once the hyperparameters are fixed. In this sense, AREGWO-TabM provides a quantitative link between biomimetic optimization, football event analysis, and training-oriented evaluation.

## 6. Conclusions

To address the limitations of the standard GWO in exploration–exploitation regulation, population diversity, elite information utilization, and stagnation recovery, this study developed an Adaptive Reinforced Elite Gray Wolf Optimizer (AREGWO). Opposition-based initialization, nonlinear convergence control, reinforced elite guidance, adaptive weak perturbation, stagnation-aware partial restart, and greedy elite inheritance are coordinated within the canonical GWO framework. The proposed method was evaluated through CEC2022 benchmark optimization, synthetic 2D constrained robotic path planning, and football pass-quality prediction. The main conclusions are summarized as follows.

(a) The CEC2022 experiments demonstrate that AREGWO provides competitive and robust optimization performance across heterogeneous benchmark landscapes. Among the nine evaluated algorithms, AREGWO ranks second in the Friedman analysis and significantly outperforms seven of the eight comparison methods in the Wilcoxon signed-rank test, while L-SHADE remains the strongest competitor. The component-wise ablation analysis further shows that the six mechanisms make complementary and problem-dependent contributions rather than relying on a single dominant strategy, supporting their coordinated use within the proposed search framework.
(b) AREGWO shows effective applicability to the tested synthetic 2D constrained path-planning scenarios. Across six randomly generated environments and repeated independent runs, it consistently generates feasible and compact collision-free routes while satisfying the prescribed safety-clearance constraints. Compared with the population-based baselines, AREGWO provides a favorable balance among route compactness, run-to-run stability, and computational efficiency. The results indicate that the proposed optimizer can effectively handle direct geometric optimization in which path length, directional variation, obstacle avoidance, and safety constraints must be considered simultaneously.
(c) Coupling AREGWO with TabM improves the prediction of the predefined football pass-quality reference score. AREGWO-TabM achieves an RMSE of 0.445, an MSE of 0.198, and an R^2^ of 0.920, corresponding to the best RMSE, MSE, and R^2^ among the evaluated neural models, although ResNet obtains a lower MAE. The TabM-based ablation further shows that AREGWO-TabM outperforms both GWO-TabM and fixed-parameter TabM, confirming the benefit of the proposed hyperparameter search strategy. This improvement is obtained at a higher offline optimization cost, and the reported predictive performance should be interpreted as consistency with the constructed reference score rather than validation against an independent tactical ground truth.

## Figures and Tables

**Table 1:** Algorithm-specific parameter settings used in the CEC2022 experiments.

Algorithm	Algorithm-Specific Parameter Settings
AREGWO	Elite weights: 0.50, 0.30, and 0.20; maximum perturbation probability: 0.20; perturbation coefficient: 0.05; stagnation threshold: 10% of the maximum number of iterations, with a minimum of 5 generations; restart proportion: 20%; restart coefficient: 0.10.
GWO	Convergence factor: linearly decreased from 2 to 0.
PSO	Cognitive coefficient: 1.5; social coefficient: 1.5; inertia weight: decreased from 0.9 to 0.4; maximum velocity: 20% of the search range.
DBO	Producer proportion: 0.20; producer-update probability threshold: 0.90; direction threshold: 0.10; producer update coefficients: 0.30 and 0.10.
WOA	Convergence factor: linearly decreased from 2 to 0; spiral constant: 1; search/spiral switching probability: 0.50.
IAGWO	Initial velocity range: ±10% of the search range; θ = 0.50; IMF parameters: 0.60, 0.02, 0.05, and 0.30.
AOGWO	Maximum jumping rate: 0.50; selective leading-opposition probability: 0.50; Lévy exponent: 1.50; Lévy scale: 0.01.
L-SHADE	Minimum population size: 4; memory size: 6; *p*-best rate: 0.11; archive rate: 1.40; initial *M_F_* = 0.50; initial *M_CR_* = 0.50; parameter sampling scale: 0.10.
DIO	Initial control parameter: 2; exploration/exploitation phase length: 25% of the total search process; exploratory branch probability: 0.50; Gaussian perturbation coefficient: 0.10.

**Table 2:** Numerical comparison results of AREGWO and the comparison algorithms on the CEC2022 benchmark functions.

Functions	Algorithms	Mean	Std.	Best	Worst	Meantime(s)
F1	AREGWO	5.32 × 10^2^	1.95 × 10^2^	3.34 × 10^2^	1.20 × 10^3^	2.65 × 10^−1^
	GWO	1.40 × 10^4^	4.89 × 10^3^	1.36 × 10^3^	2.86 × 10^4^	8.65 × 10^−2^
	PSO	1.47 × 10^3^	3.39 × 10^3^	3.00 × 10^2^	1.84 × 10^4^	6.34 × 10^−2^
	DBO	2.35 × 10^4^	8.12 × 10^3^	1.05 × 10^4^	5.05 × 10^4^	1.20 × 10^−1^
	WOA	6.61 × 10^4^	4.06 × 10^4^	2.48 × 10^4^	1.59 × 10^5^	4.09 × 10^−2^
	IAGWO	2.52 × 10^4^	3.02 × 10^3^	1.95 × 10^4^	3.08 × 10^4^	1.33 × 10^−1^
	AOGWO	7.55 × 10^4^	3.58 × 10^4^	5.74 × 10^3^	1.51 × 10^5^	1.11 × 10^−1^
	L-SHADE	3.01 × 10^2^	3.64 × 10^−4^	3.00 × 10^2^	3.02 × 10^2^	1.42× 10^0^
	DIO	2.71 × 10^4^	7.03 × 10^3^	1.31 × 10^4^	4.81 × 10^4^	6.77 × 10^−2^
F2	AREGWO	4.55 × 10^2^	1.23 × 10^1^	4.36 × 10^2^	4.77 × 10^2^	6.80 × 10^−2^
	GWO	5.11 × 10^2^	5.58 × 10^1^	4.54 × 10^2^	6.78 × 10^2^	8.84 × 10^−2^
	PSO	4.71 × 10^2^	4.50 × 10^1^	4.20 × 10^2^	6.29 × 10^2^	6.25 × 10^−1^
	DBO	4.84 × 10^2^	5.71 × 10^1^	4.08 × 10^2^	6.17 × 10^2^	1.10 × 10^−1^
	WOA	1.44 × 10^3^	4.94 × 10^2^	7.07 × 10^2^	2.96 × 10^3^	3.89 × 10^−2^
	IAGWO	2.21 × 10^3^	1.40 × 10^2^	9.13 × 10^2^	1.50 × 10^3^	1.35 × 10^−1^
	AOGWO	5.35 × 10^2^	9.56 × 10^1^	4.52 × 10^2^	9.01 × 10^2^	1.11 × 10^−1^
	L-SHADE	4.42 × 10^2^	1.58 × 10^1^	4.00 × 10^2^	4.49 × 10^2^	1.40 × 10^0^
	DIO	1.06 × 10^3^	1.55 × 10^2^	7.43 × 10^2^	1.31 × 10^3^	6.67 × 10^−2^
F3	AREGWO	6.01 × 10^2^	1.16 × 10^0^	6.00 × 10^2^	6.04 × 10^2^	1.47 × 10^−1^
	GWO	6.05 × 10^2^	2.99 × 10^0^	6.01 × 10^2^	6.12 × 10^2^	1.66 × 10^−1^
	PSO	6.03 × 10^2^	3.51 × 10^0^	6.01 × 10^2^	6.14 × 10^2^	1.39 × 10^−1^
	DBO	6.29 × 10^2^	1.10 × 10^1^	6.08 × 10^2^	6.54 × 10^2^	1.87 × 10^−1^
	WOA	6.75 × 10^2^	1.38 × 10^1^	6.49 × 10^2^	7.13 × 10^2^	1.11 × 10^−1^
	IAGWO	6.63 × 10^2^	4.40 × 10^0^	6.56 × 10^2^	6.72 × 10^2^	2.10 × 10^−1^
	AOGWO	6.13 × 10^2^	6.80 × 10^0^	6.04 × 10^2^	6.28 × 10^2^	1.88 × 10^−1^
	L-SHADE	6.01 × 10^2^	1.77 × 10^−1^	6.00 × 10^2^	6.00 × 10^2^	1.50 × 10^0^
	DIO	6.56 × 10^2^	7.52 × 10^0^	6.41 × 10^2^	6.68 × 10^2^	1.44 × 10^−1^
F4	AREGWO	8.53 × 10^2^	4.18 × 10^1^	8.14 × 10^2^	9.26 × 10^2^	9.24 × 10^−2^
	GWO	8.58 × 10^2^	2.92 × 10^1^	8.27 × 10^2^	9.29 × 10^2^	1.08 × 10^−1^
	PSO	8.46 × 10^2^	1.31 × 10^1^	8.20 × 10^2^	8.69 × 10^2^	8.78 × 10^−2^
	DBO	9.08 × 10^2^	2.51 × 10^1^	8.66 × 10^2^	9.57 × 10^2^	1.40 × 10^−1^
	WOA	9.62 × 10^2^	1.79 × 10^1^	9.25 × 10^2^	9.98 × 10^2^	6.20 × 10^−2^
	IAGWO	9.53 × 10^2^	7.96 × 10^0^	9.38 × 10^2^	9.73 × 10^2^	1.57 × 10^−1^
	AOGWO	8.91 × 10^2^	4.07 × 10^1^	8.30 × 10^2^	9.62 × 10^2^	1.34 × 10^−1^
	L-SHADE	8.16 × 10^2^	4.76 × 10^0^	8.07 × 10^2^	8.24 × 10^2^	1.39 × 10^0^
	DIO	9.67 × 10^2^	1.61 × 10^1^	9.29 × 10^2^	9.96 × 10^2^	8.64 × 10^−2^
F5	AREGWO	9.14 × 10^2^	1.80 × 10^1^	9.00 × 10^2^	9.74 × 10^2^	9.25 × 10^−2^
	GWO	1.16 × 10^3^	3.32 × 10^2^	9.25 × 10^2^	2.64 × 10^3^	1.09 × 10^−1^
	PSO	9.25 × 10^2^	2.59 × 10^1^	9.03 × 10^2^	9.96 × 10^2^	8.90 × 10^−2^
	DBO	1.87 × 10^3^	4.72 × 10^2^	1.00 × 10^3^	3.05 × 10^3^	1.56 × 10^−1^
	WOA	3.71 × 10^3^	8.97 × 10^2^	2.31 × 10^3^	5.57 × 10^3^	7.85 × 10^−2^
	IAGWO	2.73 × 10^3^	2.32 × 10^2^	2.28 × 10^3^	3.26 × 10^3^	1.62 × 10^−1^
	AOGWO	2.32 × 10^3^	1.10 × 10^3^	9.90 × 10^2^	5.26 × 10^3^	1.38 × 10^−1^
	L-SHADE	9.15 × 10^2^	2.66 × 10^1^	9.01 × 10^2^	1.04 × 10^3^	1.44 × 10^0^
	DIO	3.34 × 10^3^	6.42 × 10^2^	2.28 × 10^3^	4.40 × 10^3^	8.67 × 10^−2^
F6	AREGWO	1.00 × 10^4^	9.44 × 10^3^	2.02 × 10^3^	3.51 × 10^4^	7.37 × 10^−2^
	GWO	2.60 × 10^6^	8.02 × 10^6^	2.83 × 10^3^	3.88 × 10^7^	8.99 × 10^−2^
	PSO	1.49 × 10^6^	7.87 × 10^6^	2.03 × 10^3^	4.31 × 10^7^	6.99 × 10^−2^
	DBO	1.59 × 10^5^	4.11 × 10^5^	2.16 × 10^3^	1.49 × 10^6^	1.17 × 10^−1^
	WOA	8.63 × 10^8^	1.07 × 10^9^	3.10 × 10^7^	4.84 × 10^9^	4.48 × 10^−2^
	IAGWO	3.29 × 10^8^	1.28 × 10^8^	4.26 × 10^7^	5.97 × 10^8^	1.41 × 10^−1^
	AOGWO	3.74 × 10^6^	8.35 × 10^6^	1.09 × 10^4^	3.05 × 10^7^	1.19 × 10^−1^
	L-SHADE	1.98 × 10^3^	2.79 × 10^2^	1.85 × 10^3^	3.42 × 10^3^	1.43 × 10^0^
	DIO	3.98 × 10^8^	1.34 × 10^8^	1.80 × 10^8^	7.41 × 10^8^	7.21 × 10^−2^
F7	AREGWO	2.06 × 10^3^	4.13 × 10^1^	2.02 × 10^3^	2.21 × 10^3^	1.73 × 10^−1^
	GWO	2.08 × 10^3^	4.53 × 10^1^	2.04 × 10^3^	2.24 × 10^3^	1.89 × 10^−1^
	PSO	2.07 × 10^3^	3.13 × 10^1^	2.03 × 10^3^	2.16 × 10^3^	1.68 × 10^−1^
	DBO	2.12 × 10^3^	5.43 × 10^1^	2.04 × 10^3^	2.27 × 10^3^	2.21 × 10^−1^
	WOA	2.23 × 10^3^	6.45 × 10^1^	2.09 × 10^3^	2.39 × 10^3^	1.47 × 10^−1^
	IAGWO	2.17 × 10^3^	1.81 × 10^1^	2.13 × 10^3^	2.21 × 10^3^	2.41 × 10^−1^
	AOGWO	2.15 × 10^3^	6.76 × 10^1^	2.03 × 10^3^	2.31 × 10^3^	2.21 × 10^−1^
	L-SHADE	2.03 × 10^3^	6.49 × 10^0^	2.02 × 10^3^	2.06 × 10^3^	1.50 × 10^0^
	DIO	2.18 × 10^3^	3.09 × 10^1^	2.12 × 10^3^	2.24 × 10^3^	1.77 × 10^−1^
F8	AREGWO	2.23 × 10^3^	2.97 × 10^1^	2.22 × 10^3^	2.34 × 10^3^	2.00 × 10^−1^
	GWO	2.24 × 10^3^	4.09 × 10^1^	2.22 × 10^3^	2.35 × 10^3^	2.13 × 10^−1^
	PSO	2.27 × 10^3^	6.66 × 10^1^	2.21 × 10^3^	2.45 × 10^3^	1.94 × 10^−1^
	DBO	2.30 × 10^3^	7.17 × 10^1^	2.24 × 10^3^	2.49 × 10^3^	2.48 × 10^−1^
	WOA	2.40 × 10^3^	1.97 × 10^2^	2.33 × 10^3^	3.16 × 10^3^	1.68 × 10^−1^
	IAGWO	2.38 × 10^3^	6.99 × 10^1^	2.28 × 10^3^	2.57 × 10^3^	2.65 × 10^−1^
	AOGWO	2.36 × 10^3^	1.14 × 10^2^	2.24 × 10^3^	2.66 × 10^3^	2.46 × 10^−1^
	L-SHADE	2.22 × 10^3^	4.27 × 10^−1^	2.22 × 10^3^	2.22 × 10^3^	1.50 × 10^0^
	DIO	2.25 × 10^3^	9.69 × 10^0^	2.23 × 10^3^	2.27 × 10^3^	2.00 × 10^−1^
F9	AREGWO	2.48 × 10^3^	2.03	2.48 × 10^3^	2.49 × 10^3^	1.73 × 10^−1^
	GWO	2.50 × 10^3^	1.67 × 10^1^	2.49 × 10^3^	2.54 × 10^3^	1.87 × 10^−1^
	PSO	2.51 × 10^3^	4.53 × 10^1^	2.48 × 10^3^	2.65 × 10^3^	1.67 × 10^−1^
	DBO	2.49 × 10^3^	2.31 × 10^1^	2.48 × 10^3^	2.57 × 10^3^	2.18 × 10^−1^
	WOA	2.83 × 10^3^	1.35 × 10^2^	2.64 × 10^3^	3.20 × 10^3^	1.44 × 10^−1^
	IAGWO	2.86 × 10^3^	6.85 × 10^1^	2.71 × 10^3^	2.98 × 10^3^	2.41 × 10^−1^
	AOGWO	2.54 × 10^3^	3.76 × 10^1^	2.48 × 10^3^	2.63 × 10^3^	2.21 × 10^−1^
	L-SHADE	2.48 × 10^3^	6.86 × 10^−13^	2.48 × 10^3^	2.48 × 10^3^	1.50 × 10^0^
	DIO	2.78 × 10^3^	5.66 × 10^1^	2.66 × 10^3^	2.88 × 10^3^	1.74 × 10^−1^
F10	AREGWO	2.81 × 10^3^	7.10 × 10^2^	2.50 × 10^3^	6.10 × 10^3^	4.46 × 10^−1^
	GWO	3.03 × 10^3^	5.81 × 10^2^	2.50 × 10^3^	4.08 × 10^3^	5.23 × 10^−1^
	PSO	2.82 × 10^3^	4.01 × 10^2^	2.50 × 10^3^	3.67 × 10^3^	4.55 × 10^−1^
	DBO	2.84 × 10^3^	6.54 × 10^2^	2.50 × 10^3^	4.58 × 10^3^	6.20 × 10^−1^
	WOA	5.21 × 10^3^	1.55 × 10^3^	2.50 × 10^3^	7.20 × 10^3^	3.74 × 10^−1^
	IAGWO	2.64 × 10^3^	5.10 × 10^1^	2.56 × 10^3^	2.78 × 10^3^	2.09 × 10^−1^
	AOGWO	6.14 × 10^3^	6.10 × 10^2^	4.62 × 10^3^	7.31 × 10^3^	1.86 × 10^−1^
	L-SHADE	2.49 × 10^3^	7.00 × 10^1^	2.40 × 10^3^	2.63 × 10^3^	1.43 × 10^0^
F11	AREGWO	2.91 × 10^3^	9.67 × 10^−1^	2.90 × 10^3^	2.92 × 10^3^	6.96 × 10^−2^
	GWO	3.38 × 10^3^	2.13 × 10^2^	2.91 × 10^3^	3.92 × 10^3^	7.63 × 10^−2^
	PSO	2.90 × 10^3^	7.05 × 10^−13^	2.90 × 10^3^	2.90 × 10^3^	7.03 × 10^−1^
	DBO	2.92 × 10^3^	1.14	2.90 × 10^3^	3.00 × 10^3^	8.72 × 10^−1^
	WOA	7.69 × 10^3^	1.28 × 10^3^	4.85 × 10^3^	9.69 × 10^3^	6.16 × 10^−1^
	IAGWO	6.47 × 10^3^	3.85 × 10^2^	5.67 × 10^3^	7.18 × 10^3^	3.47 × 10^−1^
	AOGWO	9.02 × 10^3^	1.47 × 10^4^	2.98 × 10^3^	7.43 × 10^4^	3.20 × 10^−1^
	L-SHADE	2.90 × 10^3^	1.45 × 10^−12^	2.90 × 10^3^	2.90 × 10^3^	1.90 × 10^0^
	DIO	9.04 × 10^3^	4.28 × 10^2^	8.07 × 10^3^	9.83 × 10^3^	2.40 × 10^−1^
F12	AREGWO	2.95 × 10^3^	1.38	2.93 × 10^3^	2.99 × 10^3^	2.34 × 10^−1^
	GWO	2.97 × 10^3^	2.09	2.94 × 10^3^	3.03 × 10^3^	2.49 × 10^−1^
	PSO	3.02 × 10^3^	5.18 × 10^1^	2.94 × 10^3^	3.14 × 10^3^	2.30 × 10^−1^
	DBO	3.03 × 10^3^	6.75 × 10^1^	2.95 × 10^3^	3.27 × 10^3^	2.82 × 10^−1^
	WOA	3.38 × 10^3^	2.52 × 10^2^	2.98 × 10^3^	4.08 × 10^3^	2.04 × 10^−1^
	IAGWO	3.73 × 10^3^	1.19 × 10^2^	3.46 × 10^3^	3.94 × 10^3^	3.73 × 10^−1^
	AOGWO	3.00 × 10^3^	4.95 × 10^1^	2.95 × 10^3^	3.21 × 10^3^	3.54 × 10^−1^
	L-SHADE	2.95 × 10^3^	1.45 × 10^1^	2.93 × 10^3^	3.00 × 10^3^	2.00 × 10^0^
	DIO	3.34 × 10^3^	9.24 × 10^1^	3.16 × 10^3^	3.54 × 10^3^	2.94 × 10^−1^

**Table 3:** Overall statistical comparison of AREGWO and the comparison algorithms on the CEC2022 benchmark suite.

Algorithms	FriedmanMeanRank	WTL	HolmAdjustedP	RankBiserial
GWO	4.417	12/0/0	0.0039	1.000
PSO	3.750	11/0/1	0.0102	0.872
DBO	4.667	11/0/1	0.0102	0.897
WOA	8.250	12/0/0	0.0039	1.000
IAGWO	7.250	12/0/0	0.0039	1.000
AOGWO	6.500	12/0/0	0.0039	1.000
L-SHADE	1.250	2/0/10	0.0341	−0.692
DIO	6.917	12/0/0	0.0039	1.000
AREGWO	2.000	---	---	---

Note: A lower Friedman mean rank indicates better overall performance. W/T/L denotes the number of benchmark functions on which AREGWO achieves a lower/equal/higher mean final fitness than the corresponding algorithm. The Wilcoxon signed-rank tests use the 12 CEC2022 functions as paired statistical units, and the resulting *p*-values are adjusted using the Holm–Bonferroni procedure. A positive rank-biserial correlation favors AREGWO, whereas a negative value favors the comparison algorithm.

**Table 4:** Component-wise ablation results of AREGWO on representative CEC2022 functions.

Algorithm	F1	F6	F12
GWO	1.19 × 10^4^ ± 5.14 × 10^3^	2.12 × 10^6^ ± 5.38 × 10^6^	2.98 × 10^3^ ± 2.86 × 10^1^
AREGWO	5.26 × 10^2^ ± 2.41 × 10^2^	4.82 × 10^4^ ± 2.84 × 10^4^	2.94 × 10^3^ ± 6.48 × 10^0^
w/o OBI	5.92 × 10^2^ ± 2.18 × 10^2^	5.17 × 10^4^ ± 3.14 × 10^4^	2.94 × 10^3^ ± 4.11 × 10^0^
w/o NCF	6.44 × 10^2^ ± 2.32 × 10^2^	5.28 × 10^4^ ± 3.89 × 10^4^	2.94 × 10^3^ ± 7.62 × 10^0^
w/o REL	5.44 × 10^2^ ± 3.81 × 10^2^	6.16 × 10^4^ ± 3.72 × 10^4^	2.94 × 10^3^ ± 4.79 × 10^0^
w/o PERT	6.07 × 10^2^ ± 3.86 × 10^2^	6.26 × 10^4^ ± 5.80 × 10^4^	2.94 × 10^3^ ± 7.35 × 10^0^
w/o RESTART	6.09 × 10^2^ ± 2.57 × 10^2^	6.88 × 10^4^ ± 3.32 × 10^4^	2.94 × 10^3^ ± 6.39 × 10^0^
w/o GREEDY	3.98 × 10^2^ ± 4.42 × 10^1^	5.57 × 10^5^ ± 2.57 × 10^5^	2.96 × 10^3^ ± 1.57 × 10^1^

**Table 5:** Path-planning performance of the compared algorithms over 30 independent runs on six random maps.

Maps	Algorithms	Path Length (m)	Computation Time (s)	Success Rate (%)	Minimum Safety Distance (m)
Map 1	AREGWO	132.43 ± 0.02	33.062 ± 1.790	100.00	1.057 ± 0.000
	GWO	136.50 ± 3.00	88.797 ± 4.387	100.00	1.071 ± 0.012
	PSO	136.55 ± 1.95	85.011 ± 8.840	100.00	1.057 ± 0.001
	DBO	138.00 ± 2.87	77.178 ± 4.702	100.00	1.063 ± 0.013
	WOA	137.71 ± 5.40	76.615 ± 1.890	100.00	1.077 ± 0.036
	A*	134.59 ± 0.00	5.974 ± 0.359	100.00	1.089 ± 0.000
Map 2	AREGWO	137.92 ± 0.01	37.835 ± 12.046	100.00	1.057 ± 0.000
	GWO	145.83 ± 5.50	71.378 ± 7.388	100.00	1.075 ± 0.016
	PSO	141.18 ± 0.67	73.637 ± 1.906	100.00	1.057 ± 0.000
	DBO	142.94 ± 1.02	72.763 ± 0.836	100.00	1.057 ± 0.000
	WOA	143.50 ± 0.55	70.845 ± 4.620	100.00	1.066 ± 0.012
	A*	138.83 ± 0.00	10.003 ± 2.291	100.00	1.076 ± 0.000
Map 3	AREGWO	134.81 ± 0.01	29.919 ± 2.857	100.00	1.057 ± 0.000
	GWO	140.99 ± 5.72	63.611 ± 7.495	100.00	1.080 ± 0.014
	PSO	136.64 ± 0.30	74.736 ± 9.072	100.00	1.057 ± 0.000
	DBO	141.09 ± 2.77	53.909 ± 1.123	100.00	1.088 ± 0.043
	WOA	140.39 ± 4.44	58.623 ± 6.450	100.00	1.057 ± 0.000
	A*	135.94 ± 0.00	4.973 ± 0.040	100.00	1.059 ± 0.000
Map 4	AREGWO	132.14 ± 0.03	23.372 ± 0.931	100.00	1.057 ± 0.000
	GWO	140.40 ± 0.01	63.818 ± 0.527	100.00	1.061 ± 0.004
	PSO	140.22 ± 0.04	53.503 ± 6.544	100.00	1.057 ± 0.000
	DBO	137.52 ± 0.79	68.529 ± 6.059	100.00	1.060 ± 0.003
	WOA	141.59 ± 6.85	66.655 ± 6.140	100.00	1.057 ± 0.000
	A*	133.25 ± 0.00	6.707 ± 0.017	100.00	1.068 ± 0.000
Map 5	AREGWO	135.83 ± 0.01	33.684 ± 0.989	100.00	1.057 ± 0.000
	GWO	141.48 ± 0.01	63.957 ± 5.089	100.00	1.067 ± 0.013
	PSO	143.52 ± 3.01	66.679 ± 0.331	100.00	1.057 ± 0.000
	DBO	146.77 ± 1.57	64.240 ± 1.933	100.00	1.083 ± 0.009
	WOA	137.95 ± 0.76	68.235 ± 3.270	100.00	1.058 ± 0.001
	A*	137.28 ± 0.00	5.694 ± 0.113	100.00	1.101 ± 0.000
Map 6	AREGWO	133.15 ± 0.05	31.985 ± 4.183	100.00	1.057 ± 0.000
	GWO	140.07 ± 0.01	75.313 ± 4.001	100.00	1.075 ± 0.020
	PSO	138.35 ± 2.45	70.448 ± 23.354	100.00	1.057 ± 0.000
	DBO	143.44 ± 4.78	58.668 ± 7.067	100.00	1.057 ± 0.000
	WOA	140.23 ± 0.26	57.991 ± 5.028	100.00	1.088 ± 0.042
	A*	135.16 ± 0.00	6.630 ± 1.183	100.00	1.061 ± 0.000

## Data Availability

https://github.com/DYHuestc/AREGWO (accessed on 1 October 2026).
